# General Non-Markovian Quantum Dynamics

**DOI:** 10.3390/e23081006

**Published:** 2021-07-31

**Authors:** Vasily E. Tarasov

**Affiliations:** 1 Skobeltsyn Institute of Nuclear Physics, Lomonosov Moscow State University, 119991 Moscow, Russia; tarasov@theory.sinp.msu.ru; Tel.: +7-495-939-5989; 2 Faculty Information Technologies and Applied Mathematics, Moscow Aviation Institute, National Research University, 125993 Moscow, Russia

**Keywords:** fractional dynamics, open quantum systems, non-Markovian quantum dynamics, non-Hamiltonian systems, fractional calculus, general fractional calculus, nonlocality in time, 26A33, 34A08, 81S22, 26A33, 34A08, 81S22

## Abstract

A general approach to the construction of non-Markovian quantum theory is proposed. Non-Markovian equations for quantum observables and states are suggested by using general fractional calculus. In the proposed approach, the non-locality in time is represented by operator kernels of the Sonin type. A wide class of the exactly solvable models of non-Markovian quantum dynamics is suggested. These models describe open (non-Hamiltonian) quantum systems with general form of nonlocality in time. To describe these systems, the Lindblad equations for quantum observable and states are generalized by taking into account a general form of nonlocality. The non-Markovian quantum dynamics is described by using integro-differential equations with general fractional derivatives and integrals with respect to time. The exact solutions of these equations are derived by using the operational calculus that is proposed by Yu. Luchko for general fractional differential equations. Properties of bi-positivity, complete positivity, dissipativity, and generalized dissipativity in general non-Markovian quantum dynamics are discussed. Examples of a quantum oscillator and two-level quantum system with a general form of nonlocality in time are suggested.

## 1. Introduction

The dynamics of open quantum systems (OQS) is usually described by Markovian equation for quantum observables and quantum states [1,2,3,4,5]. The most general explicit form of such equations were first proposed by Gorini, Kossakowski, Sudarshan, and Lindblad in 1976 [6,7,8]. Quantum mechanics of OQS has been actively developing (see [1,2,3,4,5,9, and reviews [10,11,12]). The Markovian dynamics of OQS is characterized by non-standard properties that lead to a change in the usual relations and approaches, for example, such as the uncertainty relation for open quantum systems [12,13], path integrals [5] (pp. 475–485) and [14], pure stationary states [5] (pp. 453–462) and [15,16], and quantum computer with mixed states [5] (pp. 487–520) and [17]. We can say that the theory of open quantum systems is the most general type of modern quantum mechanics such as fundamental theory [5]. Note that OQS are non-Hamiltonian quantum systems, that is, it is not enough to specify the Hamilton operator to describe the dynamics of such systems [5]. This theory has great practical importance for the creation of quantum computers and quantum informatics due to the fact that the influence of the environment on the process of quantum computation, which is realized by the dynamics of the quantum systems of qubits.

Currently, modern quantum mechanics is faced with the question of the most general form of the equations that describe the non-Markovian dynamics of quantum systems. A general form of non-Markovian character of quantum processes can be interpreted as nonlocality in time, or memory as its special form. Nonlocality in time means a dependence of the dynamics of observables and states at the current moment of time in the history of the system’s behavior on a finite interval in the past.

Attempts to construct a non-Markovian theory of open quantum systems with non-locality in time have been actively undertaken in recent years (see, for example, reviews [18,19,20], and articles [21,22,23,24,25] and references therein). Most approaches to the formulation of non-Markovian quantum dynamics were not related to fractional calculus and mathematical theory of equations with derivatives and integrals of non-integer orders (see [26,27,28,29,30,31,32]). Equations with fractional derivatives and integrals of non-integer orders are a powerful tool to take into account nonlocality in space and time in physics [33,34]. It should be emphasized that the characteristic property of fractional differential operators of non-integer orders is nonlocality in space and time [35,36].

For the first time, the use of fractional derivatives and integrals of non-integer order with respect to time to describe non-Markovian dynamics of OQS (in the form of power-law fading memory) was proposed in [37] (see Chapter 20 in book [37] (pp. 477–482) and [38,39,40]). Exact solutions of generalized Lindblad equations, which describe non-Markovian quantum dynamics, were derived in these works.

Fractional powers of Lindblad superoperators were first defined and used to describe OQS in [5] (pp. 433–444), (see also [37] (pp. 458–464, 468–477), and [41,42,43]). Solutions of equations, which describe non-Markovian quantum dynamics, were derived in works [5,37,41,42]. The generalization, which used the Grunvald–Letnikov fractional derivatives, is suggested in [44].

We also note some other formulations of the non-Markovian quantum dynamics by using fractional calculus: the non-Markovian dynamics of OQS with time-dependent parameters [39,45], relativistic open classical systems [46], and quantum systems with memory [47].

Note that the non-Markovian quantum dynamics is also considered in the framework of generalizations of the Schrodinger and Heisenberg equations (see [5,37,43,48,49,50] (pp. 457–466), respectively).

In fractional calculus, nonlocality is described by the kernel of the operators, which are fractional integrals (FI) and fractional derivatives (FD) of non-integer orders. To describe dynamical systems with various types of nonlocality in space and time, we can use operators with various types of kernels. Therefore it is important to have a general fractional calculus that allows us to describe nonlocality in the most general form.

The concept of general fractional calculus (GFC) has been suggested by Anatoly N. Kochubei in his work [51] in 2011 (see also [52,53,54]) out of connection with quantum theory. In works in [51,52] the general fractional derivatives (GFD) and general fractional integral (GFI) are defined. For these operators, the general fundamental theorems were proven in [51,52]. This approach to GFC is based on the concept of kernel pairs, which was proposed by N.Ya. Sonin (1849–1915) in their 1884 article [55] (see also [56]. Note that the name “Sonin” [57] is mistakenly used in French transliteration as “N. Sonine” from French journals [55]). Then, very important results in constructing the GFC were derived by Yuri Luchko in 2021 [58,59,60]. In works [58,59], GFD and GFI of arbitrary order are suggested, and the general fundamental theorems for the GFI and GFDs were proven. Operational calculus for equations with general fractional derivatives was proposed in [60]. The general fractional calculus was also developed in works [61,62,63,64,65,66,67,68,69,70,71,72] devoted to mathematical aspects and some applications in classical physics.

The first application of the general fractional study is proposed in this article. The general fractional calculus is suggested as a mathematical basis to formulate the non-Markovian quantum dynamics. In the framework of the general approach, it was assumed and implied to obtain, first of all, general results that do not depend on specific types (particular implementations) of nonlocality and kernels [72]. In the general approach to non-Markovian quantum dynamics, all research and results should be related to the general form of nonlocality, operator kernels of almost all types (a wide set of operator kernels). In this paper, we consider general fractional operators with kernels that belong to the Sonin set. The general form of non-Markovian dynamics of open quantum systems is described by the equations with general fractional derivatives and integrals [58,59]. The exact solutions of these equations were derived by using the general operational calculus proposed in [60].

## 2. Markovian Dynamics of Quantum States and Observables

In this section, we will briefly describe the basics of Markovian quantum dynamics for fixing concepts and notations.

Quantum states can be described by density operators ρ that are normalized ($Tr\left[\rho \right]\;=1$), self-adjoint ($\rho^{†}=\rho$), positive (ρ≥0) operators. All these properties must be conserved in the time evolution $S_{t}:\;\rho \;\to \;\rho_{t}$. Therefore to describe Markovian quantum dynamics in general form, we should find such a time evolution for the density operators $\rho_{t}=S_{t}\rho$, which satisfy the following conditions for all $t\in \;\left(0,\infty \right)$.

(1) The self-adjoint condition

(1) $${\left(\rho_{t}\right)}^{†}=\rho_{t},\;(\mathrm{forallt}>0).$$

(2) The positivity condition

(2) $$\rho_{t}>0,\;(\mathrm{forallt}>0).$$

(3) The normalization condition

(3) $$Tr\left[\rho_{t}\right]\;=1,\;(\mathrm{forallt}>0).$$

If we consider the Markovian dynamics, then the semigroup condition is used

(4) $$S_{t}\;S_{\tau }=S_{t+\tau }$$

for all t,τ≥0, and the condition $S_{0}\rho =\rho$ is represented as $S_{t}\left(\rho \right)\;\to \rho$ at t→0 in the trace norm.

The Markovian quantum dynamics of quantum observables $A_{t}\;=\;\Phi_{t}\left(A\right)$ is described by the dual dynamical maps $\Phi_{t}$, where the duality is represented by the condition

(5) $$Tr\left[A\rho_{t}]\;=Tr[A_{t}\rho \right],\;(\mathrm{forallt}>0).$$

Let A be an algebra of bounded operators (for example, $C^{*}$-algebra, or algebra of $B\left(H\right)$ of bounded operators on the Hilbert space H).

Normalization condition (3) means that $\Phi_{t}\left(I\right)\;=I$, where I is the identity operator.

Positivity condition (2) means that $\Phi_{t}\left(A^{†}A\right)\;\geq \;0$. The positivity condition is usually replaced by the complete positivity condition for the dynamical maps $\Phi_{t}$:

(6) $$\sum_{k=1}^{n}\sum_{l=1}^{n}A_{k}^{†}\Phi_{t}\left(B_{k}^{†}\;B_{l}\right)A_{l}\geq 0$$

for all $A_{j},B_{j}\in A$, j=1,…n and all n∈ℕ.

To describe the Markovian dynamics of quantum observables, the semigroup condition is used

(7) $$\Phi_{t}{\;\Phi }_{\tau }={\;\Phi }_{t+\tau }$$

for all t,τ≥0, and the condition $\Phi_{0}\left(A\right)\;=A$ is represented as $\Phi_{t}\left(A\right)\;\to \;A$ at t→0 in ultraweak operator topology. The ultraweak operator topology can be defined as the topology induced on A by the set of all seminorms of the form

(8) $${∥A∥}_{\sigma ,\rho }\;=\;\left|Tr\left[\rho \;A\right]\right|.$$

For the complete positive semigroup $\left\{\Phi_{t}:\;t\in \;\left(0,\infty \right)\right\}$, for which $\Phi_{t}\left(I\right)\;=I$ and $\Phi_{t}\left(A\right)\;\to \;A$ ultraweakly at t→0, there exists a superoperator L for which

(9) $$\frac{d}{dt}\Phi_{t}\left(A\right)\;=\;L\Phi_{t}\left(A\right)$$

holds for all $A\in D\left(L\right)$, where $D\left(L\right)$ is a ultraweakly dense domain of A. The superoperator L is called the generator of the semigroup $\Phi_{t}$. The dual generator Λ of the semigroup $S_{t}$ is connected with L through the equation

(10) $$Tr\left[\left(LA\right)\;\rho \right]\;=\;Tr\left[A\;\left(\Lambda \rho \right)\right].$$

For superoperator Λ, we have

(11) $$\frac{d}{dt}S_{t}\left(\rho \right)\;=\;\Lambda S_{t}\left(\rho \right).$$

The bounded superoperators are defined on the normed operator space A: The superoperator L is called bounded, if

(12) $$∥L\left(A\right){∥}_{A}\;\leq \;c{∥A∥}_{A}$$

for some constant c and all A∈A. Inequality (12) means that L transforms norm bounded sets of A into the norm bounded sets. The least value of c equal to

(13) $$∥L∥\;=\;\underset{A\neq 0}{\sup}\frac{∥L\left(A\right){∥}_{A}}{∥A{∥}_{A}},$$

is called the norm of the superoperator L. If A is a normed space, and L is a bounded superoperator, then

(14) ∥Λ∥ = ∥L∥.

Let us define the class of real superoperators: A real superoperator is a superoperator L on A, such that

(15) $${\left[L\left(A\right)\right]}^{†}=L\left(A^{†}\right)$$

for all $A\in D\left(L\right)\;\subset A$ and $A^{†}\in D\left(L\right)$, where $A^{†}$ be adjoint of A∈A. If L is a real superoperator, then Λ is real.

The Lindblad proved theorem defines the structure of the superoperators L and Λ. This theorem is proved for the completely positive dynamical semigroup $\Phi_{t}$, which is norm continuous

(16) $$\underset{t\to 0}{\lim}∥\Phi_{t}\left(A\right)\;-\;A{∥}_{A}\;=0.$$

Equation (16) is a more restrictive condition than ultraweak continuity. For such semigroup. the superoperator L is bounded.

For completely positive maps $\Phi_{t}$, the bi-positivity condition

(17) $$\Phi_{t}\left(A^{†}\;A\right)\;\geq {\;\Phi }_{t}\left(A^{†}\right){\;\Phi }_{t}\left(A\right)$$

holds for all t>0 with equality at t=0. The differentiation of inequality (17) gives at t=0 the property of the superoperator

(18) $$L\left(A^{†}\;A\right)\;\geq \;L\left(A^{†}\right)\;A+A^{†}\;L\left(A\right)$$

for all A∈A. The bounded superoperators L, which satisfy conditions $L\left(I\right)\;=0$, $L\left(A^{†}\right)\;={\left(LA\right)}^{†}$ and inequality (18), are called dissipative. The real superoperator L is called completely dissipative, if

(19) $$L\left(A_{k}^{†}\;A_{l}\right)\;\geq \;L\left(A_{k}^{†}\right)\;A_{l}+A_{k}^{†}\;L\left(A_{l}\right),$$

where k,l=1,…,n, for all n∈ℕ and all $A_{k}^{†},\;A_{l},\;\left(A_{k}^{†}A_{l}\right)\;\in D\left(L\right)\;\subset A$, where $A_{k}^{†},\;\left(A_{k}^{†}A_{l}\right)\;\in D\left(L\right)$.

The Lindblad theorem gives the most general form of completely dissipative superoperators L.

The Lindblad theorem states [7] that the superoperator L is completely dissipative and ultraweakly continuous if and only if it has the form

(20) $$LA\;=\;-\frac{1}{i\hbar }\left[H,A\right]\;+\;\frac{1}{2\hbar }\sum_{k=1}^{\infty }\left(V_{k}^{†}\;\left[A,V_{k}\right]\;+\;\left[V_{k}^{†},A\right]V_{k}\right),$$

where $H^{†}=H\in A$, $V_{k}\in A$, $\sum_{k}V^{†}\;V_{k}\;\in \;A$ The dual superoperator Λ has the form

(21) $$\Lambda \rho \;=\;\frac{1}{i\hbar }\left[H,\rho \right]\;+\;\frac{1}{2\hbar }\sum_{k=1}^{\infty }\left(\left[V_{k}\;\rho ,V_{k}^{†}\right]\;+\;\left[V_{k},\;\rho \;V_{k}^{†}\right]\right).$$

Equations (20) and (21) give explicit forms of most general Markovian dynamics of quantum observables and states.

The quantum Markovian equation, which are also called the Lindblad equations, are written in the form

(22) $$\frac{dA\left(t\right)}{dt}=-\frac{1}{i\hbar }\left[H,A\left(t\right)\right]\;+\;\frac{1}{2\hbar }\sum_{k=1}^{\infty }\left(V_{k}^{†}\left[A\left(t\right),V_{k}\right]\;+\;\left[V_{k}^{†},A\left(t\right)\right]V_{k}\right),$$

(23) $$\frac{d\rho \left(t\right)}{dt}\;=\;\frac{1}{i\hbar }\left[H,\rho \left(t\right)\right]\;+\;\frac{1}{2\hbar }\sum_{k=1}^{\infty }\left(\left[V_{k}\rho \left(t\right),V_{k}^{†}\right]\;+\;\left[V_{k},\rho \left(t\right)V_{k}^{†}\right]\right),$$

where $A\left(t\right)$ is a quantum observable; $\rho \left(t\right)$ is a quantum state; H is the Hamiltonian operator; and $V_{k}$ are the Lindblad operators [5]. Equations (22) and (23) are the standard time-local (memoryless) Markovian equations for quantum observables and states [6,7,8].

If $V_{k}=0$ for all k∈ℕ, then Equations (22) and (23) give the standard Heisenberg equation and von Neumann equation, respectively. In this case, Equations (22) and (23) describe Markovian dynamics of Hamiltonian quantum systems without nonlocality in time.

## 3. General Non-Markovian Dynamics of Quantum Observables and States

For the description of non-Markovian quantum processes, we can take into account nonlocality in time, which means that the behavior of the quantum observable $A\left(t\right)$ (or state $\rho \left(t\right)$) and its derivatives may depend on the history of previous changes of this operator. To describe this type of behavior, we cannot use differential equations of integer orders.

To take into account nonlocality in time (non-Markovality) in open quantum systems in work [37] (Chapter 20) and [38,39], it has been proposed to use the derivatives of non-integer orders instead of integer-order derivatives with respect to time.

In this section, we proposed using general fractional calculus and general fractional derivatives (GFD) as mathematical tools to allow us to take into account the general nonlocality in time for non-Markovian quantum processes.

### 3.1. Generalization of Lindblad Equation for Quantum Observables

The Lindblad equation for quantum observables is described by the operator differential equation of the first order

(24) $$\frac{dA\left(t\right)}{dt}=L\;A\left(t\right),$$

where the superoperator L is defined by expression (20). Equation (24) can be written in the integral form

(25) $$A\left(t\right)\;-\;A\left(0\right)\;=\;\int_{0}^{t}\;d\tau \;L\;A\left(\tau \right).$$

The nonlocality in time can be taken into account by using an integral kernel in generalization of Equation (25) in the form

(26) $$A\left(t\right)\;-\;A\left(0\right)\;=\;\int_{0}^{t}d\tau \;M\left(t-\tau \right)\;L\;A\left(\tau \right),$$

where the function $M\left(t-\tau \right)$ describes nonlocality in time. If $M\left(t\right)\;=1$ for all $t\;\in \;\left(0,\infty \right)$, then Equation (26) gives standard Equation (24), which describes the Markovian quantum dynamics.

Obviously, not all kernels $M\left(t-\tau \right)$ can describe nonlocality in time. The nonlocality requirement can be formulated as follows. If integral Equation (26) can be written as a differential equation of an integer order (or a finite system of such equations), then the process, which is described by Equation (26), is local in time. The first obvious example of such “local” kernel is

(27) $$M\left(t\right)\;=h_{\alpha }\left(t\right)\;=\frac{t^{\alpha -1}}{\Gamma \left(\alpha \right)}$$

with positive integer values of α=n∈ℕ. Examples of “local” kernels are also the probability density functions of the exponential distribution and the gamma distribution with integer shape parameters (the Erlang distribution) [73,74].

Note that kernel (27) with non-integer values of α>0 define “non-local” kernel of the Riemann–Liouville and Caputo fractional operators [29].

Let us assume that the functions $M\left(t\right)$ belongs to the space $C_{-1,0}\left(0,\infty \right)$, and suppose that there exists a function $K\left(t\right)\;\in C_{-1,0}\left(0,\infty \right)$, such that the Laplace convolution of these functions is equal to one for all $t\in \;\left(0,\infty \right)$. The function $X\left(t\right)$ belongs to the space $C_{-1,0}\left(0,\infty \right)$, if this function can be represented in the form $X\left(t\right)\;=t^{p}\;Y\left(t\right)$, where −1<p<0, and $Y\left(t\right)\;\in \;c\;\left[0,\infty \right)$.

**Definition** **1.** *The functions $M\left(t\right),K\left(t\right)$ are a Sonin pair of kernels, if the following conditions are satisfied*

*(1)*  *The Sonin condition for the kernels $M\left(t\right)$ and*$K\left(t\right)$*requires that the relations*
  (28) $$\int_{0}^{t}M\left(t-\tau \right)\;K\left(\tau \right)\;d\tau =1$$
  holds for all $t\in \;\left(0,\infty \right)$.

*(2)*  *The functions $M\left(t\right),K\left(t\right)$ belong to the space*
  (29) $$C_{-1,0}\left(0,\infty \right)\;=\{X\left(t\right):\;X\left(t\right)\;=t^{p}\;Y\left(t\right),\;\;t>0,\;-1<p<0,\;\;Y\left(t\right)\;\in \;C\left[0,\infty \right)\}.\;$$
  *The set of such Sonin kernels is denoted by $S_{-1}$.*

If the kernel pair $\left(M\left(t\right),K\left(t\right)\right)$ belongs to Sonin set $S_{-1}$ [59], the kernel $K\left(t\right)$ is called the associated kernel to $M\left(t\right)$. Note that if $K\left(t\right)$ is the associated kernel to $M\left(t\right)$, then $M\left(t\right)$ is the associated kernel to $K\left(t\right)$. Therefore, if $\left(M\left(t\right),K\left(t\right)\right)$ belongs to set $S_{-1}$, then $I_{\left(M\right)}^{t}$, $D_{\left(K\right)}^{t}$, and $I_{\left(K\right)}^{t}$, $D_{\left(M\right)}^{t}$ can be used as the general fractional integrals (GFI) and general fractional derivatives (GFD).

To define GFI and GFD, we used Luchko’s approach to general fractional calculus, which is proposed in [58,59].

**Definition** **2.** *Let $M\left(t\right)\;\in S_{-1}$ and* $X\left(t\right)\;\in C_{-1}\left(0,\infty \right)\;=C_{-1,\infty }\left(0,\infty \right)$ *. The general fractional integral (GFI) with the kernel $M\left(t\right)\;\in C_{-1,0}\left(0,\infty \right)$ is the operator on the space* $C_{-1}\left(0,\infty \right)$ *, that is*

(30) $$I_{\left(M\right)}^{t}:\;\;C_{-1}\left(0,\infty \right)\;\to C_{-1}\left(0,\infty \right),\;$$

*that is defined by the equation*

(31) $$I_{\left(M\right)}^{t}\left[\tau \right]X\left(\tau \right)\;=\left(M\;*\;X\right)\left(t\right)\;=\;\int_{0}^{t}d\tau \;M\left(t-\tau \right)\;X\left(\tau \right).$$

If the functions $M\left(t\right)$ and $K\left(t\right)$ belong to the Sonin set, then we can define general fractional derivatives $D_{\left(K\right)}^{t}$ and $D_{\left(K\right)}^{t,*}$ that are associated with GFI $I_{\left(M\right)}^{t}$.

**Definition** **3.** *Let $M\left(t\right),K\left(t\right)\;\in S_{-1}$ and* $X\left(t\right)\;\in C_{-1}^{1}\left(0,\infty \right)$ *, i.e., $X^{\left(1\right)}\in C_{-1}\left(0,\infty \right)$. The general fractional derivatives (GFD) with kernel* $K\left(t\right)\;\in C_{-1,0}\left(0,\infty \right)$ *, which is associated with GFI (31), is defined as*

(32) $$D_{\left(K\right)}^{t,*}\left[\tau \right]X\left(\tau \right)\;=\;\left({K\;*\;X}^{\left(1\right)}\right)\left(t\right)\;=\;\int_{0}^{t}d\tau \;K\left(t-\tau \right){\;X}^{\left(1\right)}\left(\tau \right)$$

*for $t\in \;\left(0,\infty \right)$. The GFD $D_{\left(K\right)}^{t}$ is defined by the equation*

(33) $$D_{\left(K\right)}^{t}\left[\tau \right]X\left(\tau \right)\;=\frac{d}{\mathrm{dt}}\left(K\;*\;X\right)\left(t\right)\;=\;\int_{0}^{t}\frac{d}{\mathrm{dt}}d\tau \;K\left(t-\tau \right)\;X\left(\tau \right)$$

*for $t\in \;\left(0,\infty \right)$.*

As proven in [58,59], operators (32) and (33) are connected (see Equation (47) in Definition 4 of [58] (p. 8)) by the equation

(34) $$D_{\left(K\right)}^{t,*}\left[\tau \right]X\left(\tau \right)\;=D_{\left(K\right)}^{t}\left[\tau \right]X\left(\tau \right)\;-\;K\left(t\right)\;X\left(0\right).$$

The proposed GFI and GFD can be used to formulate non-Markovian dynamics in the general form, where the nonlocality in time is described by the kernel pairs that belong to the Sonin set $S_{-1}$.

If $\left(M\left(t\right),K\left(t\right)\right)\;\in S_{-1}$, then Equation (26) can be written through the GFI with kernel $M\left(t\right)$ as

(35) $$A\left(t\right)\;-\;A\left(0\right)\;=I_{\left(M\right)}^{t}\left[\tau \right]\;L\;A\left(\tau \right).$$

The action of the GFD $D_{\left(K\right)}^{s}\left[t\right]$ with kernel $K\left(t\right)$, which is associated to $M\left(t\right)$, in Equation (35), gives

(36) $$D_{\left(K\right)}^{s}\left[t\right]\;A\left(t\right)\;-\;A\left(0\right)D_{\left(K\right)}^{s}\left[t\right]\;1=D_{\left(K\right)}^{s}\left[t\right]\;I_{\left(M\right)}^{t}\left[\tau \right]\;L\;A\left(\tau \right).$$

For the right-hand side of Equation (36), we use the first fundamental theorem of GFC (see Theorem 3 of [58] (p. 9)). This theorem states that the equation

(37) $$D_{\left(K\right)}^{s}\left[t\right]\;I_{\left(M\right)}^{t}\left[\tau \right]\;f\left(\tau \right)\;=f\left(s\right),$$

holds for $f\left(t\right)\;\in C_{-1}\left(0,\infty \right)$.

Using Equation (37) and the equality

(38) $$D_{\left(K\right)}^{s}\left[t\right]\;1\;=\;\frac{d}{ds}\int_{0}^{s}dt\;K\left(s-t\right)\;1\;=\;\frac{d}{ds}\int_{0}^{s}d\xi \;K\left(\xi \right)\;=\;K\left(s\right),$$

Equation (36) is written in the form

(39) $$D_{\left(K\right)}^{s}\left[t\right]\;A\left(t\right)\;-\;A\left(0\right)\;K\left(t\right)\;=L\;A\left(s\right).$$

The left-hand side of Equation (39) can be expressed through GFD $D_{\left(K\right)}^{s,*}$ by using Equation (34) in the form

(40) $$D_{\left(K\right)}^{s}\left[t\right]\;A\left(t\right)\;-\;A\left(0\right)K\left(s\right)\;=D_{\left(K\right)}^{s,*}\left[t\right]\;A\left(t\right)$$

for $A\left(t\right)\;\in C_{-1}^{1}\left(0,\infty \right)$ (see also Equations (47) and (49) in [58] (p. 8)).

As a result, Equation (36) can be written as

(41) $$D_{\left(K\right)}^{t,*}\left[\tau \right]\;A\left(\tau \right)\;=L\;A\left(t\right),$$

where L is the Lindblad superoperator (the Lindbladian, quantum Liouvillian). Equation (41) describes non-Markovian dynamics of quantum observables in the general form, where the kernel belongs to the Sonin set $S_{-1}$.

As a result, we proved the following theorem.

**Theorem** **1.** *The integral equation*

(42) $$A\left(t\right)\;-\;A\left(0\right)\;=\;\int_{0}^{t}d\tau \;M\left(t-\tau \right)\;L\;A\left(\tau \right),$$

*where $M\left(t\right)\;\in C_{-1,0}\left(0,\infty \right)$ and* $A\left(t\right)\;\in C_{-1}^{1}\left(0,\infty \right)$ *, L is the Lindblad superoperator (20), which can be represented in the form*

(43) $$D_{\left(K\right)}^{t,*}\left[\tau \right]\;A\left(\tau \right)\;=L\;A\left(t\right),\;$$

*if there exists the kernel $K\left(t\right)\;\in C_{-1,0}\left(0,\infty \right)$*, which is associated with*$M\left(t\right)$ such that the pair* $\left(M\left(t\right),K\left(t\right)\right)$ *belongs to the Sonin set $S_{-1}$.*

### 3.2. Generalization of Lindblad Equation for Quantum States

Equation (23) is the standard memoryless Markovian quantum master equation [6,7,8]. Equation (23) can be written in the integral form

(44) $$\rho \left(t\right)\;-\;\rho \left(0\right)\;=\;\int_{0}^{t}\left(\Lambda \;\rho \right)\left(\tau \right)\;d\tau ,$$

The nonlocality in time can be taken into account by using an integral kernel in Equation (44) in the form

(45) $$\rho \left(t\right)\;-\;\rho \left(0\right)\;=\;\int_{0}^{t}M\left(t\;-\;\tau \right)\;\left(\Lambda \;\rho \right)\left(\tau \right)\;d\tau .$$

If $M\left(t\right)\;=1$ for $t\in \;\left(0,\infty \right)$, then Equation (45) gives standard Equation (44). In general, the kernel $M\left(t-\tau \right)$ can be used to describe non-Markovian quantum dynamics.

As a result, the non-Markovian master equation for quantum states takes the form

(46) $$D_{\left(K\right)}^{t,*}\left[\tau \right]\;\rho \left(\tau \right)\;=\;\Lambda \;\rho \left(t\right),$$

if $\left(M\left(t\right),K\left(t\right)\right)\;\in S_{-1}$. Equation (46) describes non-Markovian dynamics of quantum states in the general form, where the kernel $K\left(t\right)$ belongs to the Sonin set $S_{-1}$.

### 3.3. Luchko Functions

Let us consider the triple $R_{-1}=\;\left(C_{-1}\left(0,\infty \right),*,+\right)$, where the multiplication ∗ is the Laplace convolution and + the standard addition of functions. The triple $R_{-1}$ is a commutative ring without divisors of zero [58,75].

The solutions of Equations (41) and (46) can be derived by using the Luchko operational calculus [60]. To describe the solution, we give the following theorem and define the Luchko function.

**Theorem** **2.** *Let $M\left(t\right)$ be a kernel from the Sonin set* $S_{-1}$ *and the power series*

(47) $$f\left(x,\lambda \right)\;=\;\sum_{j=0}^{\infty }{\;\lambda }^{j-1}{\;x}^{j}$$

*has non-zero convergence radius r. Then the convolution series*

(48) $$F\left(M,\lambda ,t\right)\;=\;\sum_{j=0}^{\infty }{\;M}^{*,j}\left(t\right){\;\lambda }^{j-1}$$

*is convergent for all $t\in \;\left(0,\infty \right)$ and the function* $F\left(x,\lambda ,t\right)$ *belongs to the ring $R_{-1}$.*

Theorem 2 is proven in [60] (see Theorem 4.4 in [60] (p. 359) and comments on [60] (p. 360)).

**Definition** **4.** *Let $M\left(t\right)$ be a kernel from the Sonin set*$S_{-1}$*, and $M^{*,j}\left(t\right)$ is the convolution*j*-power:*

(49) $$M^{*,j}\left(t\right)\;:=\;\left(M_{1}\;*\;\ldots \;*\;M_{j}\right)\left(t\right),\;$$

*where $M_{k}\left(t\right)\;=\;M\left(t\right)$ for all k=1, …, j, and*$t\in \;\left(0,\infty \right)$.

*Then, the function*

(50) $$F\left(M,\lambda ,t\right)\;=\;\sum_{j=0}^{\infty }{\;M}^{*,j}\left(t\right){\;\lambda }^{j-1}$$

*will be called the first Luchko function.*

Let us give examples of the first Luchko function [60] (p. 361).

(1) For the Sonin kernel
  (51) $$M\left(t\right)\;=h_{\alpha }\left(t\right)\;=\frac{t^{\alpha -1}}{\Gamma \left(\alpha \right)},\;M^{*,j}\left(t\right)\;=h_{j\alpha }\left(t\right),$$
  the first Luchko function has the form
  (52) $$F\left(M,\lambda ,t\right)\;=\;\sum_{j=1}^{\infty }\lambda^{j-1}h_{j\alpha }\left(t\right)\;=t^{\alpha -1}\sum_{j=1}^{\infty }\frac{\lambda^{j}\;t^{j\alpha }}{\Gamma \left(j\alpha +\alpha \right)}=t^{\alpha -1}E_{\alpha ,\alpha }\left(\lambda \;t^{\alpha }\right).$$

Here, $E_{\alpha ,\beta }\left(z\right)$ is the two-parameters Mittag–Leffler function [76] that is defined as

(53) $$E_{\alpha ,\beta }\left(z\right)\;:=\;\sum_{k=0}^{\infty }\frac{z^{k}}{\Gamma \left(\alpha k+\beta \right)},$$

where α>0, β∈ℝ (or β∈ℂ).

(2) For the Sonin kernel
  (54) $$M\left(t\right)\;=h_{\alpha ,\beta }\left(t\right)\;=h_{\alpha }\left(t\right)e^{-\beta t},M^{*,j}\left(t\right)\;=h_{j\alpha ,\beta }\left(t\right).$$
  the first Luchko function takes the form
  (55) $$F\left(M,\lambda ,t\right)\;=\;\sum_{j=1}^{\infty }\lambda^{j-1}h_{j\alpha ,\beta }\left(t\right)\;=e^{-\beta t}t^{\alpha -1}\sum_{j=1}^{\infty }\frac{\lambda^{j}\;t^{j\alpha }}{\Gamma \left(j\alpha +\alpha \right)}=e^{-\beta t}t^{\alpha -1}E_{\alpha ,\alpha }\left(\lambda \;t^{\alpha }\right).$$
(3) For the Sonin kernel
  (56) $$M\left(t\right)\;=h_{1-\beta +\alpha }\left(t\right)\;+\;h_{1-\beta }\left(t\right),$$
  where 0<α<β<1, the first Luchko function is given as
  (57) $$F\left(M,\lambda ,t\right)\;=\;\sum_{j=1}^{\infty }\lambda^{j-1}\sum_{i=0}^{j}\left(\begin{array}{l} j\\ i \end{array}\right)h_{i\alpha +j\left(1-\beta \right)}\left(t\right)\;=\;\frac{1}{\lambda t}\sum_{j=1}^{\infty }\sum_{l_{1}+l_{2}=j}\frac{j!}{l_{1}!l_{2}!}\frac{{\left(\lambda t^{1-\beta +\alpha }\right)}^{l_{1}}{\left(\lambda t^{1-\beta }\right)}^{l_{2}}}{\Gamma \left(l_{1}\left(1-\beta +\alpha \right)\;+\;l_{2}\left(1-\beta \right)\right)}=\;\frac{1}{\lambda t}E_{\left(1-\beta ,1-\beta +\alpha \right),0}\left(\lambda t^{1-\beta },\lambda t^{1-\beta +\alpha }\right),$$
  where $E_{\left(1-\beta ,1-\beta +\alpha \right),0}$ is the binomial (multinomial) Mittag–Leffler function [29] (p. 49) and [77], which is defined as
  (58) $$E_{\left(\alpha_{1},\ldots ,\alpha_{m}\right),\beta }\left(z_{1},\ldots ,z_{m}\right)\;:=\sum_{j=0}^{\infty }\sum_{l_{1}+\cdots +l_{m}=j}\frac{j!}{\prod_{i=1}^{m}l_{i}!}\frac{\prod_{i=1}^{m}z_{i}^{li}}{\Gamma \left(\sum_{i=1}^{m}\alpha_{i}l_{i}+\beta \right)}$$

Using the first Luchko function $F\left(M,\lambda ,t\right)$ and the kernel $K\left(t\right)$ that is associated with $M\left(t\right)$, we can define the second Luchko function.

**Definition** **5.** *Let the functions $\left(M\left(t\right),K\left(t\right)\right)$ belong to the Sonin set* $S_{-1}$ *, and the first Luchko function is*

(59) $$F\left(M,\lambda ,t\right)\;=\sum_{j=1}^{\infty }{\;M}^{*,j}\left(t\right){\;\lambda }^{j-1}.$$

*Then, the function*

(60) $$L\left(M,\lambda ,t\right)\;:=\;\int_{0}^{t}d\tau \;K\left(t-\tau \right)F\left(M,\lambda ,\tau \right)\;={\;I}_{\left(K\right)}^{t}\left[\tau \right]F\left(M,\lambda ,\tau \right),$$

*will be called the second Luchko function.*

Note that Equation (60) contains the GFI with kernel $K\left(t\right)$ rather than kernel $M\left(t\right)$.

The second Luchko function (60) is used [60] in solution of equations with GFD that is defined by the kernel $K\left(t\right)$ associated with the kernel $M\left(t\right)$ of the GFI.

To derive solutions of equations with GFD, Yu. Luchko proposed the general operational calculus [60]. Theorem 5.1 of [60] (p. 366) proves that the solution of the equation

(61) $$D_{\left(K\right)}^{t,*}\left[\tau \right]\;A\left(\tau \right)\;=\lambda \;A\left(t\right),$$

where $A\left(t\right)\;\in C_{-1}^{1}\left(0,\infty \right)$ is expressed through the second Luchko function $L\left(M,\lambda ,\tau \right)$.

If $\left(M\left(t\right),K\left(t\right)\right)\;\in S_{-1}$, then $F\left(M,\lambda ,t\right)\;\in C_{-1}\left(0,\infty \right)$ and $L\left(M,\lambda ,t\right)\;\in C_{-1}\left(0,\infty \right)$. Therefore, these Luchko functions belong to the ring $R_{-1}$. Thess statements are based on the fact that GFI $I_{\left(K\right)}^{t}\left[\tau \right]$ is the operator on $C_{-1}\left(0,\infty \right)$ (see Equation (30) and reference [60]).

Note that the second Luchko function (60) can be considered as independent of the kernel $K\left(t\right)$ since the Sonin condition $\left(K\;*\;M\right)\left(t\right)\;=\left\{1\right\}$ is satisfied for all $t\in \;\left(0,\infty \right)$.

Using the superoperator form of the first Luchko function $F\left(M,\lambda ,t\right)$ and the second Luchko function $L\left(M,\lambda ,t\right)$, we can propose solutions of equations for non-Markovian open quantum systems with nonlocality in time.

### 3.4. General Form of Solutions for Non-Markovian Equations

Let L be a bounded superoperator on the normed operator algebra A, in other words,

(62) λ := ∥L∥ <∞,

and $L^{0}=L_{I}$ is the unit superoperator ($L_{I}\;A=A$ for all A∈A). The superoperator power series

(63) $$F\left(x,L\right)\;:=\;\sum_{j=1}^{\infty }x^{j}\;L^{j-1}$$

converges in norm and the radius of convergence is equal to

(64) $$r=\lambda^{-1}=\;∥L{∥}^{-1},$$

if L is the bounded superoperator on the normed operator algebra A.

Using Theorem 2, we can state that the series

(65) $$F\left(M,L,t\right)\;:=\;\sum_{j=1}^{\infty }M^{*,j}\left(t\right)\;L^{j-1}$$

converges in norm for all $t\in \;\left(0,\infty \right)$, if $M\left(t\right)\;\in C_{-1}\left(0,\infty \right)$ and L is the bounded superoperator.

The solution of Equation (41) can be expressed through the superoperator (65).

To obtain the solution of the general non-Markovian equation for quantum observable A∈A, we will use Theorem 5.1 of [60] (p. 366).

**Theorem** **3.** *Let $A\left(t\right)\;\in C_{-1}^{1}\left(0,\infty \right)$, the pair $\left(M\left(t\right),K\left(t\right)\right)$ is Sonin pair from $S_{-1}$, and L is a bounded superoperator on A. Then the initial value problem*

(66) $$D_{\left(K\right)}^{t,*}\left[\tau \right]\;A\left(\tau \right)\;=L\;A\left(t\right),\;\;\;\;A\left(0\right)\;=A_{0}\;$$

*has the unique solution*

(67) $$A\left(t\right)\;=\;\Phi_{\left(M\right)}^{t}A_{0},$$

*where*

(68) $$\Phi_{\left(M\right)}^{t}\;=\;L\left(M,L,t\right)\;=\;\int_{0}^{t}\;d\tau \;K\left(t-\tau \right)\;F\left(M,L,\tau \right)\;={\;I}_{\left(K\right)}^{t}\left[\tau \right]\;F\left(M,L,\tau \right).$$

The proof of this theorem is based on Theorem 5.1, which was proven in [60] (pp. 366).

**Remark** **1.** *Note that superoperator $\Phi_{\left(M\right)}^{t}$ is independent of the kernel $K\left(t\right)$ due to the Sonin condition*

(69) $$\left(M\;*\;K\right)\left(t\right)\;=\;\left\{1\right\}.\;$$

*Using condition (69), we get the convolution of kernel $K=K\left(t\right)$ and $L=F\left(M,L,t\right)$ in the form*

(70) $$\Phi_{\left(M\right)}^{t}\;=\;\left(K\;*\;L\right)\left(t\right)\;=\;\sum_{j=1}^{\infty }\left(K\;*\;M^{*,j}\right)\left(t\right)\;L^{j-1}\;=\;\;\sum_{j=1}^{\infty }\;\left(K\;*\;M\;*\;M^{*,j-1}\right)\left(t\right)\;L^{j-1}\;=\;\;\sum_{j=1}^{\infty }\left(\left\{1\right\}\;*\;M^{*,j-1}\right)\left(t\right)\;L^{j-1}\;=\;L_{I}\;+\;\left(\left\{1\right\}\;*\;\sum_{j=2}^{\infty }\left(M^{*,j-1}\;L^{j-1}\right)\right)\left(t\right)\;=\;\;L_{I}\;+\;\left(\left\{1\right\}\;*\;\sum_{j=1}^{\infty }\left(M^{*,j}\;L^{j}\right)\right)\left(t\right),$$

*where $L^{0}=L_{I}$ is the unit superoperator ($L_{I}\;A=A$ for all A∈A).*

*Therefore the map $\Phi_{\left(M\right)}^{t}$ is written as*

(71) $$\Phi_{\left(M\right)}^{t}\;=\int_{0}^{t}\;K\left(t-\tau \right)\;F\left(M,L,\tau \right)\;={\;L}_{I}\;+\;\int_{0}^{t}\left(\sum_{j=1}^{\infty }{\;M}^{*,j}\left(\tau \right){\;L}^{j}\right)d\tau .$$

In the next section, we give some examples of the general non-Markovian Equation (66) and solutions (67).

### 3.5. Example of General Non-Markovian Dynamics

Let us consider some special cases of the proposed general non-Markovian equations and its solutions that are derived by the Luchko operational calculus [60].

(1) In the first example, we consider the Sonin pairs of the kernels
  (72) $$M\left(t\right)\;=h_{\alpha }\left(t\right)\;=\frac{t^{\alpha -1}}{\Gamma \left(\alpha \right)},\;K\left(t\right)\;=h_{1-\alpha }\left(t\right),$$
  where t>0, and 0<α<1. In this case, the GFD $D_{\left(K\right)}^{t,*}$ is the Caputo derivative $D_{C,0+}^{\alpha }$ with $\alpha \in \;\left(0,1\right)$. Then
  (73) $$\Phi_{\left(M\right)}^{t}\;=\;L_{I}\;+\;\left\{1\right\}\;*\;\sum_{j=1}^{\infty }\;h_{j\alpha }\left(t\right)\;L^{j}\;=\;L_{I}+\;\sum_{j=1}^{\infty }h_{j\alpha +1}\left(t\right)\;L^{j}\;=\;E_{\alpha }\left(t^{\alpha }\;L\right),$$
  where the Mittag–Leffler function $E_{\alpha }\left(z\right)\;=E_{\alpha ,1}\left(z\right)$ [76]. The fractional differential equation
  (74) $$D_{\left(K\right)}^{t,*}\left[\tau \right]A\left(\tau \right)\;=LA\left(t\right),\;A\left(0\right)\;=A_{0},$$
  has the solution
  (75) $$A\left(t\right)\;=E_{\alpha }\left(t^{\alpha }\;L\right)\;A_{0}.$$

This type of non-Markovian quantum dynamics was first described in [37] (Chapter 20) and [38,39].

(2) In the second example, we consider the Sonin pairs of the kernels
  (76) $$M\left(t\right)\;=h_{\alpha ,\beta }\left(t\right)\;=h_{\alpha }\left(t\right)\;e^{-\beta t},\;K\left(t\right)\;=h_{1-\alpha }\left(t\right)\;e^{-\beta t}+\frac{\beta^{\alpha }}{\Gamma \left(1-\alpha \right)}\;\gamma \left(1-\alpha ,\beta t\right),$$
  where t>0, $\alpha \in \;\left(0,1\right)$, β>0, and $\gamma \left(\beta ,t\right)$ is the incomplete gamma function
  (77) $$\gamma \left(\beta ,t\right)\;=\;\int_{0}^{t}\tau^{\beta -1}e^{-\tau }\;d\tau .$$

The GFD has the form

(78) $$D_{\left(K\right)}^{t,*}\left[\tau \right]\;A\left(\tau \right)\;=\;\int_{0}^{t}\left(h_{1-\alpha }\left(\tau \right)\;e^{-\beta \tau }\;+\;\frac{\beta^{\alpha }}{\Gamma \left(1-\alpha \right)}\;\gamma \left(1-\alpha ,\beta \tau \right)\right)\;A^{\left(1\right)}\left(t-\tau \right)\;d\tau .$$

The superoperator $\Phi_{\left(M\right)}^{t}$ is written as

(79) $$\Phi_{\left(M\right)}^{t}\;=\;\left(K\;*F\left(M,L,\tau \right)\right)\left(t\right)\;=\;\;\left(\left(h_{1-\alpha ,\beta }+\beta \left\{1\right\}\;*\;h_{1-\alpha ,\beta }\right),\sum_{j=1}^{\infty }h_{j\alpha ,\beta }\;L^{j-1}\right)\left(t\right)\;=\;\;\sum_{j=1}^{\infty }h_{\left(j-1\right)\alpha +1,\beta }\left(t\right)L^{j-1}\;+\;\beta \left(\left\{1\right\}\;*\sum_{j=1}^{\infty }h_{\left(j-1\right)\alpha +1,\beta }\;L^{j-1}\right)\left(t\right)\;=\;\;e^{-\beta t}\;E_{\alpha }\left(t^{\alpha }\;L\right)\;+\;\beta \;\int_{0}^{t}d\tau \;e^{-\beta \tau }E_{\alpha }\left(\tau^{\alpha }\;L\right),$$

where we use $\left(h_{\alpha ,\beta }\;*\;h_{\gamma ,\beta }\right)\;=h_{\alpha +\gamma ,\beta }.$

For nonlocality (76), the equation of non-Markovian dynamics

(80) $$D_{\left(K\right)}^{t,*}\left[\tau \right]A\left(\tau \right)\;=LA\left(t\right),\;A\left(0\right)\;=A_{0},$$

has the solution

(81) $$A\left(t\right)\;=\;\left(e^{-\beta t}\;E_{\alpha }\left(t^{\alpha }\;L\right)\;+\;\beta \int_{0}^{t}\;d\tau \;e^{-\beta \tau }E_{\alpha }\left(\tau^{\alpha }\;L\right)\right)\;A_{0}.$$

(3) In the third example, we consider the Sonin pairs of the kernels
  (82) $$M\left(t\right)\;=h_{1-\beta +\alpha }\left(t\right)\;+\;h_{1-\beta }\left(t\right),\;K\left(t\right)\;=t^{\beta -1}\;E_{\alpha ,\beta }\left(-t^{\alpha }\right),$$
  where 0<α<β<1. In this case, we use the GFD that has the Mittag–Leffler function in the kernel
  (83) $$D_{\left(K\right)}^{t,*}\left[\tau \right]\;A\left(\tau \right)\;=\;\int_{0}^{t}\;d\tau \;\tau^{\beta -1}\;E_{\alpha ,\beta }\left(-\tau^{\alpha }\right)\;A^{\left(1\right)}\left(t-\tau \right).$$

Then, the non-Markovian dynamics is described by the superoperator

(84) $$\Phi_{\left(M\right)}^{t}\;=\;L_{I}\;+\;\left\{1\right\}\;*\sum_{j=1}^{\infty }M^{*,j}\left(t\right)L^{j}=\;L_{I}\;+\;\left\{1\right\}\;*\sum_{j=1}^{\infty }L^{j}\sum_{i=0}^{j}\left(\begin{array}{l} j\\ i \end{array}\right)h_{i\alpha +j\left(1-\beta \right)}\left(t\right)\;=\;E_{\left(1-\beta ,1-\beta +\alpha \right),1}\left(t^{1-\beta }\;L,t^{1-\beta +\alpha }\;L\right),$$

where the function $E_{\left(1-\beta ,1-\beta +\alpha \right),1}$ is a special case (binomial) of the multinomial Mittag–Leffler function [77]. The equation with GFD (83) and initial condition

(85) $$D_{\left(K\right)}^{t,*}\left[\tau \right]A\left(\tau \right)\;=LA\left(t\right),\;A\left(0\right)\;=A_{0},$$

has the solution

(86) $$A\left(t\right)\;=E_{\left(1-\beta ,1-\beta +\alpha \right),1}\left(t^{1-\beta }L,t^{1-\beta +\alpha }\;L\right)\;A_{0}.$$

These examples of equations and their solutions describe the non-Markovian dynamics of quantum observables.

## 4. Properties of Non-Markovian Quantum Dynamical Maps

### 4.1. Violation of Semigroup Property for Non-Markovian Maps

The non-Markovian maps $\Phi_{t}^{\left(M\right)}\left(\alpha \right)$, t>0 describe dynamics of open quantum systems with power-law memory. The superoperator L can be considered as a generator of the one-parameter groupoid $\Phi_{t}^{\left(M\right)}\left(\alpha \right)$ on an operator algebra of quantum observables:

$$D_{\left(K\right)}^{t,*}\left[\tau \right]{\;\Phi }_{\tau }^{\left(M\right)}=L{\;\Phi }_{\tau }^{\left(M\right)}.$$

The set $\left\{\Phi_{t}^{\left(M\right)}\;|t\;>\;0\right\}$, is called a quantum dynamical groupoid [5,38]. Note that the following properties are realized

$$\Phi_{t}^{\left(M\right)}\;I=I,$$

$${\left(\Phi_{t}^{\left(M\right)}\;A\right)}^{†}=\Phi_{t}^{\left(M\right)}\;A$$

for self-adjoint operators A ($A^{†}=A$), and

$$\underset{t\to 0+}{\lim}\Phi_{t}^{\left(M\right)}=L_{I},$$

where $L_{I}$ is an identity superoperator ($L_{I}\;A=A$). As a result, the non-Markovian map $\Phi_{t}^{\left(M\right)}$, t>0, are real and unit preserving maps on the operator algebra of quantum observables.

For $M\left(t\right)\;=h_{\alpha }\left(t\right)$ and $K\left(t\right)\;=h_{1-\alpha }\left(t\right)$ with 0<α≤1, the GFD is the Caputo fractional derivative of the order α, and the non-Markovian map has the form

$$\Phi_{t}^{\left(M\right)}=E_{\alpha }\left[t^{\alpha }\;L\right]$$

that is described in [37,38], where $E_{\alpha }\left[z\right]$ is the Mittag–Leffler function [76]. For α=1, we have

$$\Phi_{t}^{\left(M\right)}=E_{1}\left[t\;L\right]\;=\;\exp\left\{t\;L\right\}\;={\;\Phi }_{t}.$$

The superoperators $\Phi_{t}$ form a semigroup such that

$$\Phi_{t}{\;\Phi }_{s}={\;\Phi }_{t+s},(t,s>0),{\;\Phi }_{0}=L_{I}.$$

This property holds since

$$\exp\left\{t\;L\right\}\;\exp\left\{s\;L\right\}\;=\;\exp\left\{\left(t+s\right)\;L_{V}\right\}.$$

For α∈ℕ, we have the violation of the semigroup property [78,79,80]:

$$E_{\alpha }[t^{\alpha }\;L]\;E_{\alpha }[s^{\alpha }\;L]\;\neq \;E_{\alpha }[\left(t+s{)}^{\alpha }\;L\right].$$

Therefore, the semigroup property is not satisfied for non-Markovian dynamics

$$\Phi_{t}^{\left(M\right)}{\;\Phi }_{s}^{\left(M\right)}\;\neq {\;\Phi }_{t}^{\left(M\right)},(t,s>0).$$

As a result, the non-Markovian maps $\Phi_{t}^{\left(M\right)}$ cannot form a semigroup. This property is a characteristic property, which means that we have a quantum process with non-locality in time. The maps $\Phi_{t}^{\left(M\right)}$ describe the quantum dynamics of open systems with non-locality in time. This non-locality in time means that their present value of quantum observable (or quantum state) of quantum system $A\left(t\right)\;=\Phi_{t}^{\left(M\right)}A_{0}$ depends on all past values of $A\left(\tau \right)$ for $\tau \in \;\left[0,t\right]$.

### 4.2. Some Properties of Markovian Maps

In this section, we will briefly describe the properties of Markovian maps $\Phi_{t}$ and the superoperator L, for the convenience of generalizations to non-Markovian dynamics.

#### 4.2.1. Bi-Positivity and Dissipativity in Markovian Theory

The Markovian quantum dynamics is described by the map

(87) $$\Phi_{t}\;:=\;\sum_{n=0}^{\infty }h_{n+1}\left(t\right)\;L^{n},$$

where

$$h_{n+1}\left(t\right)\;=\frac{t^{n}}{\Gamma \left(n+1\right)}=\frac{t^{n}}{n!}.$$

We will assume that $D\left(L\right)\;=A$ to simplify the description. To this purpose, we will also consider the bi-positivity condition instead of complete positivity condition.

The bi-positivity condition for the Markovian map $\Phi_{t}$ can be considered in the form

(88) $$\Phi_{t}\left(A\;A^{†}\right)\;\geq {\;\Phi }_{t}\left(A\right){\;\Phi }_{t}\left(A^{†}\right),$$

which should be satisfied for all t>0 and A∈A.

The importance of condition (88) is due to the fact that it leads to the positive condition

$$\Phi_{t}\left(A\;A^{†}\right)\;\geq \;0,$$

for all A∈A, if $\Phi_{t}\left(A^{†}\right)\;={\left(\Phi_{t}\left(A\right)\right)}^{†}$. Let us prove this statement. If $L\left(A^{†}\right)\;={\left(L\left(A\right)\right)}^{†}$, then

$$\Phi_{t}\left(A^{†}\right)\;=\;{\left(\Phi_{t}\left(A\right)\right)}^{†},$$

and

$$\Phi_{t}\left(A\right){\;\Phi }_{t}\left(A^{†}\right)\;={\;\Phi }_{t}\left(A\right)\;{\left(\Phi_{t}\left(A\right)\right)}^{†}={\left|\Phi_{t}\left(A\right)\right|}^{2}\;\geq \;0.$$

As a result, we obtain

$$\Phi_{t}\left(A\;A^{†}\right)\;\geq \;{\left|\Phi_{t}\left(A\right)\right|}^{2}\;\geq \;0.$$

If the real superoperator L is completely dissipative, for which the inequality

(89) $$L\left(A_{k}^{†}\;A_{l}\right)\;\geq \;L\left(A_{k}^{†}\right)\;A_{l}+A_{k}^{†}\;L\left(A_{l}\right)$$

is satisfied for all $A_{k},A_{l}\in A$, then the quantum Markovian map map $\Phi_{t}$ is completely positive, if $\Phi_{t}\left(A^{†}\right)\;={\left(\Phi_{t}\left(A\right)\right)}^{†}$ for all A∈A. This statement can be proved similarly by using the following transformations

$$\sum_{k=1}^{n}\sum_{l=1}^{n}B_{k}^{†}\Phi_{t}\left(A_{k}^{†}\;A_{l}\right)B_{l}\geq \;\left(\sum_{k=1}^{n}B_{k}^{†}\Phi_{t}\left(A_{k}^{†}\right)\right)\left(\sum_{l=1}^{n}\Phi_{t}\left(A_{l}\right)B_{l}\right)\;=\;{\left(\sum_{l=1}^{n}\Phi_{t}\left(A_{l}\right)B_{l}\right)}^{†}\left(\sum_{l=1}^{n}\Phi_{t}\left(A_{l}\right)B_{l}\right)\;=\;{|\sum_{l=1}^{n}\Phi_{t}\left(A_{l}\right)B_{l}|}^{2}\;\geq \;0.$$

Let us consider two approaches to find a condition that the real superoperator L must satisfy in order for the bi-positivity condition to be satisfied for all t>0 in the Markovian quantum dynamics, and a possibility to generalize these approaches to the general non-Markovian maps.

#### 4.2.2. Markovian Case: First Approach

The condition on L to have the bi-positivity of maps $\Phi_{t}$ is usually obtained by differentiating inequality (88) with respect to time

(90) $$\frac{d}{dt}\Phi_{t}\left(A\;A^{†}\right)\;\geq \;\frac{d}{dt}\left(\Phi_{t}\left(A\right){\;\Phi }_{t}\left(A^{†}\right)\right)$$

and using the standard Leibniz rule

(91) $$\frac{d}{dt}\left(\Phi_{t}\left(A\right){\;\Phi }_{t}\left(A^{†}\right)\right)\;=\frac{d}{dt}\left(\Phi_{t}\left(A\right)\right){\;\Phi }_{t}\left(A^{†}\right)\;+\;\Phi_{t}\left(A\right)\;\frac{d}{dt}\left(\Phi_{t}\left(A^{†}\right)\right).$$

The Markovian equations for quantum observables

$$\frac{d}{dt}\Phi_{t}\left(A\right)\;=L{\;\Phi }_{t}\left(A\right)\;={\;\Phi }_{t}\left(LA\right)$$

and Equation (91) allow us to get inequality (90) in the form

(92) $$\Phi_{t}\left(L\left(A\;A^{†}\right)\right)\;\geq {\;\Phi }_{t}\left(L\left(A\right)\right){\;\Phi }_{t}\left(A^{†}\right)\;+\;\Phi_{t}\left(A\right){\;\Phi }_{t}\left(L\left(A^{†}\right)\right).$$

In the limit t → 0, we get the condition

(93) $$L\left(A\;A^{†}\right)\;\geq \;L\left(A\right)\;A^{†}+A\;L\left(A^{†}\right).$$

Unfortunately, this approach cannot be used for equations with fractional derivatives and GFD because the standard Leibniz rule (the product rule) is violated

(94) $$D_{\left(K\right)}^{t,*}\left[\tau \right]\left(\Phi_{\tau }\left(A\right){\;\Phi }_{\tau }\left(A^{†}\right)\right)\;\neq \;D_{\left(K\right)}^{t,*}\left[\tau \right]\left(\Phi_{\tau }\left(A\right)\right){\;\Phi }_{t}\left(A^{†}\right)\;+\;\Phi_{t}\left(A\right)\;D_{\left(K\right)}^{t,*}\left[\tau \right]\left(\Phi_{t}\left(A^{†}\right)\right).$$

For example, in the non-Markovian quantum theory, which was proposed in [37] (pp.477–482) and [38,39], the Sonin pair of kernels is used in the form

$$M\left(t\right)\;=h_{\alpha }\left(t\right)\;=\frac{t^{\alpha -1}}{\Gamma \left(\alpha \right)},\;K\left(t\right)\;=h_{1-\alpha }\left(t\right)\;=\frac{t^{-\alpha }}{\Gamma \left(1-\alpha \right)},$$

with 0<α<1. In this case, the GFI and GFD are the Riemann–Liouville fractional integral and the Caputo fractional derivative. The generalized Leibniz rule (see Theorem 3.17 in [30] (p. 59)) has the form

$$D_{\left(h_{1-\alpha }\right)}^{t,*}\left[\tau \right]\;\left(f\left(\tau \right)\;g\left(\tau \right)\right)\;=\;h_{1-\alpha }\left(t\right)\;\left(f\left(t\right)\;-\;f\left(0\right)\right)\;g\left(0\right)\;+\;f\left(t\right)\;\left(D_{\left(h_{1-\alpha }\right)}^{t,*}\left[\tau \right]\;g\left(\tau \right)\right)\;+\;\sum_{k=1}^{\infty }\left(\begin{array}{l} \alpha \\ k \end{array}\right)\;f^{\left(k\right)}\left(t\right)\;I_{\left(h_{k-\alpha }\right)}^{t}\left[\tau \right]\;g\left(\tau \right),$$

where $\left(\begin{array}{l} \alpha \\ k \end{array}\right)\;:=\frac{\alpha \left(\alpha -1\right)\ldots \left(\alpha -k+1\right)}{k!}.$

The violation of the standard Leibniz rule is a characteristic property of fractional derivatives of non-integer order [81].

For GFD, there is no rule for differentiating the product in the general case. Therefore, we should use another approach to derive the conditions on the superoperator L. For Markovian dynamics, another method of obtaining the condition on L can be used, and this method can be generalized to the case of non-Markovian quantum theory.

#### 4.2.3. Markovian Case: Second Approach

Let us consider the bi-positivity condition in the form

(95) $$\langle \Phi_{t}\left(A\;A^{†}\right)\rangle \;\geq \;\langle \Phi_{t}\left(A\right){\;\Phi }_{t}\left(A^{†}\right)\rangle ,$$

which should be satisfied for all t>0 and all A∈A, where the average value of the quantum observable $A\left(t\right)\;=\Phi_{t}\left(A\right)$ is defined as

$$\langle \Phi_{t}\left(A\right)\rangle =Tr\left[\rho {\;\Phi }_{t}\left(A\right)\right].$$

Substitution of expression (87) into inequality (95) gives

(96) $$\sum_{n=0}^{\infty }h_{n+1}\left(t\right)\;\langle L^{n}\left(A\;A^{†}\right)\rangle \;\geq \;\sum_{m=0}^{\infty }\sum_{k=0}^{\infty }h_{m+1}\left(t\right)\;h_{k+1}\left(t\right)\;\langle L^{m}\left(A\right)\;L^{k}\left(A^{†}\right)\rangle ,$$

where we use the linearity of the average value. Using n=m+k, and

$$h_{m+1}\left(t\right)\;h_{k+1}\left(t\right)\;=\frac{t^{m}}{\Gamma \left(m+1\right)}\;\frac{t^{k}}{\Gamma \left(k+1\right)}\;=\;\frac{\Gamma \left(m+k+1\right)}{\Gamma \left(m+1\right)\;\Gamma \left(k+1\right)}\;\frac{t^{m+k}}{\Gamma \left(m+k+1\right)}\;=\;\left(\begin{array}{l} m+k\\ k \end{array}\right)\;h_{m+k+1}\left(t\right)\;=\;\left(\begin{array}{l} n\\ k \end{array}\right)\;h_{n+1}\left(t\right)$$

inequality (96) is represented as

(97) $$\sum_{n=o}^{\infty }h_{n+1}\left(t\right)\;\langle L^{n}\left(A\;A^{†}\right)\rangle \;\geq \;\sum_{n=o}^{\infty }h_{n+1}\left(t\right)\;\sum_{k=o}^{n}\left(\begin{array}{l} n\\ k \end{array}\right)\;\langle L^{n-k}\left(A\right)\;L^{k}\left(A^{†}\right)\rangle .$$

Since the bi-positivity condition must be satisfied for all t>0, we obtain

(98) $$\langle L^{n}\left(A\;A^{†}\right)\rangle \;\geq \;\sum_{k=0}^{n}\left(\begin{array}{l} n\\ k \end{array}\right)\;\langle L^{n-k}\left(A\right)\;L^{k}\left(A^{†}\right)\rangle ,$$

which should be satisfied for all n∈ℕ. For n=1, inequality (98) has the form

(99) $$\langle L\left(A\;A^{†}\right)\rangle \;\geq \;\langle L\left(A\right)\;A^{†}+A\;L\left(A^{†}\right)\rangle ,$$

which should be satisfied for all A∈A.

The bounded superoperators L, which satisfy conditions $L\left(I\right)\;=0$, $L\left(A^{†}\right)\;={\left(LA\right)}^{†}$ and inequality (99), are called dissipative.

Inequality (99) for real superoperator L is a necessary and sufficient condition in order for the Markovian quantum map $\Phi_{t}$ to have a bi-positivity property.

**Theorem** **4.** *Let L be real superoperator, which satisfies the dissipativity condition*

(100) $$L\left(A\;A^{†}\right)\;\geq \;L\left(A\right)\;A^{†}+A\;L\left(A^{†}\right)\;$$

*for all A∈A.*

*Then, the Markovian map*$\Phi_{t}$*satisfies the bi-positive condition*

$$\Phi_{t}\left(A\;A^{†}\right)\;\geq \;\Phi_{t}\left(A\right)\;\Phi_{t}\left(A^{†}\right)$$

*for all*t>0*and all*A∈A.

**Proof.**  Using the series representation of $\Phi_{t}\left(A\;A^{†}\right)$ and inequality

(101) $$L\left(A^{†}\;A\right)\;\geq \;L\left(A^{†}\right)\;A+A^{†}\;L\left(A\right)$$

we get

$$\Phi_{t}\left(AA^{†}\right)\;=\;\sum_{n=0}^{\infty }h_{n+1}\left(t\right)L^{n}\left(AA^{†}\right)\;\geq \;\sum_{n=0}^{\infty }\sum_{k=0}^{n}\left(\begin{array}{l} n\\ k \end{array}\right)h_{n+1}\left(t\right)L^{n-k}\left(A\right)L^{k}\left(A^{†}\right)\;=\;\sum_{m=0}^{\infty }\sum_{k=0}^{\infty }\left(\begin{array}{l} m+k\\ k \end{array}\right)h_{m+k+1}\left(t\right)L^{m}\left(A\right)L^{k}\left(A^{†}\right).$$

Using

$$\left(\begin{array}{l} n\\ k \end{array}\right)\;h_{m+k+1}\left(t\right)\;=\frac{\Gamma \left(m+k+1\right)}{\Gamma \left(m+1\right)\Gamma \left(k+1\right)}\;h_{m+k+1}\left(t\right)\;=\;\frac{\Gamma \left(m+k+1\right)}{\Gamma \left(m+1\right)\Gamma \left(k+1\right)}\;\frac{t^{m+k}}{\Gamma \left(m+k+1\right)}\;=\;\frac{t^{m}\;t^{k}}{\Gamma \left(m+1\right)\Gamma \left(k+1\right)}\;=h_{m+1}\left(t\right)\;h_{k+1}\left(t\right)$$

we get

$$\Phi_{t}\left(A\;A^{†}\right)\;\geq \;\sum_{m=0}^{\infty }\sum_{k=0}^{\infty }h_{m+1}\left(t\right)\;h_{k+1}\left(t\right)\;L^{m}\left(A\right)\;L^{k}\left(A^{†}\right)\;=\;\;\left(\sum_{m=0}^{\infty }\;h_{m+1}\left(t\right)\;L^{m}\left(A\right)\right)\left(\sum_{k=0}^{\infty }\;h_{k+1}\left(t\right)\;L^{k}\left(A^{†}\right)\right)\;={\text{ Φ}}_{t}\left(A\right){\text{ Φ}}_{t}\left(A^{†}\right).$$

 □

### 4.3. General Non-Markovian Maps: Bi-Positivity and Complete Positivity

In this section, we will consider the bi-positivity condition instead of the complete positivity condition to simplify the description and proofs. Condition of complete positivity proved similar to the proofs for bi-positivity, and conditions for complete dissipativity of the superoperator L are written analogously to conditions for general dissipativity. We will also assume that $D\left(L\right)\;=A$ to simplify the description.

Let us give the definition for the non-Markovian quantum maps that are described in the paper.

**Definition** **6.** *Let the pair of kernels $\left(M\left(t\right),\;K\left(t\right)\right)$ belongs to the Sonin set $S_{-1}$, where the Sonin condition has the form*

$$\left(K\;*\;M\right)\left(t\right)\;=\;\left\{1\right\},\;$$

*and* L *is real Lindblad superoperator.*

*Then, the one-parameter superoperator*

(102) $$\Phi_{t}^{\left(M\right)}\left(A\right)\;=\;\sum_{j=1}^{\infty }\left({K\;*\;M}^{*,j}\right)\left(t\right){\;L}^{j-1}\;=\;\sum_{n=0}^{\infty }M_{n+1}\left(t\right){\;L}^{n},$$

*where*

(103) $$M_{n+1}\left(t\right)\;:=\;\left({K\;*\;M}^{*,n+1}\right)\left(t\right)\;=\;\left(\left\{1\right\}{\;*\;M}^{*,n}\right)\left(t\right)\;=\int_{0}^{t}\;M^{*,n}\left(\tau \right)\;d\tau ,$$

*will be called the general non-Markovian quantum map.*

For the general non-Markovian quantum dynamics, the bi-positivity property of the map $\Phi_{t}^{\left(M\right)}$ can be described in the following form.

**Definition** **7.** *The bi-positivity property of the general non-Markovian quantum dynamical map $\Phi_{t}^{\left(M\right)}$ can be defined in the form of the inequality*

(104) $$\langle \Phi_{t}^{\left(M\right)}\left(A\;A^{†}\right)\rangle \;\geq \;\langle \Phi_{t}^{\left(M\right)}\left(A\right)\;\Phi_{t}^{\left(M\right)}\left(A^{†}\right)\rangle ,\;$$

*which holds for all t>0 and all*A∈A. *The average value of the quantum observable $A\left(t\right)\;=\Phi_{t}^{\left(M\right)}\left(A\right)$ is defined as*

$$\langle \Phi_{t}^{\left(M\right)}\left(A\right)\rangle =Tr\left[\rho \;\Phi_{t}^{\left(M\right)}\left(A\right)\right].\;$$

**Lemma** **1.** *Let the general non-Markovian map $\Phi_{t}^{\left(M\right)}$ be bi-positive and*

$${\left(\Phi_{t}^{\left(M\right)}\left(A\right)\right)}^{†}=\Phi_{t}^{\left(M\right)}\left(A^{†}\right)\;$$

*for all t>0 and all A∈A.*

*Then, the positivity condition*

$$\langle \Phi_{t}^{\left(M\right)}\left(A\;A^{†}\right)\rangle \;\geq \;0$$

*holds for all t>0 and all* A∈A *.*

**Proof.**  The following equalities hold

$$\langle \Phi_{t}^{\left(M\right)}\left(A\right)\;{\left(\Phi_{t}^{\left(M\right)}\left(A\right)\right)}^{†}\rangle =\langle A\left(t\right)\;A^{†}\left(t\right)\rangle =\langle {\left|A\left(t\right)\right|}^{2}\rangle \;\geq \;0$$

for all t>0 and for all A∈A. Therefore, bi-positivity condition (104) leads to the positivity

$$\langle \Phi_{t}^{\left(M\right)}\left(A\;A^{†}\right)\rangle \;\geq \;\langle \Phi_{t}^{\left(M\right)}\left(A\right){\;\Phi }_{t}^{\left(M\right)}\left(A^{†}\right)\rangle =\;\langle \Phi_{t}^{\left(M\right)}\left(A\right)\;{\left(\Phi_{t}^{\left(M\right)}\left(A\right)\right)}^{†}\rangle =\langle |\Phi_{t}^{\left(M\right)}\left(A\right){|}^{2}\rangle \;\geq \;0$$

in the form

$$\langle \Phi_{t}^{\left(M\right)}\left(A\;A^{†}\right)\rangle \;\geq \;0.$$

 □

**Definition** **8.** *The complete positivity condition of the general non-Markovian quantum dynamical map $\Phi_{t}^{\left(M\right)}$ can be defined in the form*

(105) $$\sum_{k=1}^{n}\sum_{l=1}^{n}\langle B_{k}^{†}\Phi_{t}^{\left(M\right)}\left(A_{k}^{†}{\;A}_{l}\right)B_{l}\rangle \;\geq \;0,$$

*which holds for all t>0, and for all*$A_{j},B_{j}\in A$, j=1,…n*and all n∈ℕ.*

**Lemma** **2.** *Let the general non-Markovian map $\Phi_{t}^{\left(M\right)}$ satisfy the complete positivity condition, and*

$${\left(\Phi_{t}^{\left(M\right)}\left(A\right)\right)}^{†}=\Phi_{t}^{\left(M\right)}\left(A^{†}\right)\;$$

*for all*t>0*and all*A∈A.

*Then the positivity condition*

(106) $$\langle \Phi_{t}^{\left(M\right)}\left(A\;A^{†}\right)\rangle \;\geq \;0.$$

*holds for all t>0.*

**Proof.**  Using $A_{k}=A$ and $B_{k}=I$, inequality (105) takes form (106). □

#### 4.3.1. From Bi-Positivity to General Dissipativity

Let us find a condition that the real superoperator L must satisfy in order to bi-positivity condition (104) to be satisfied for all t>0 for the general non-Markovian quantum maps.

This required condition is given by the following theorem.

**Theorem** **5.** *Let the bi-positivity condition*

(107) $$\langle \Phi_{t}^{\left(M\right)}\left(A\;A^{†}\right)\rangle \;\geq \;\langle \Phi_{t}^{\left(M\right)}\left(A\right)\;\Phi_{t}^{\left(M\right)}\left(A^{†}\right)\rangle \;$$

*be satisfied for all t>0 for the general non-Markovian map*

(108) $$\Phi_{t}^{\left(M\right)}\left(A\right)\;=\;\sum_{n=0}^{\infty }M_{n+1}\left(t\right){\;L}^{n},$$

*where*

(109) $$M_{n}\left(t\right)\;>\;0\;$$

*for all*n∈ℕ*and all*t>0.

*Then real superoperator* L *satisfies the inequality*

(110) $$\langle L^{n}\left(A,A^{†}\right)\rangle \geq \sum_{k=1}^{n}E_{k}^{n}\left(K,M\right)\langle L^{n-k}\left(A\right)L^{k}\left(A^{†}\right)\rangle ,$$

*for all* n∈ℕ *and all t>0, where*

(111) $$E_{k}^{n}\left(K,M\right)\;:=\frac{M_{n-k+1}\left(t\right)\;M_{k+1}\left(t\right)}{M_{n+1}\left(t\right)}.\;$$

**Proof.**  Substitution of expression (102) in the right side of inequality (104) gives

(112) $$\langle \Phi_{t}^{\left(M\right)}\left(A\right){\;\Phi }_{t}^{\left(M\right)}\left(A^{†}\right)\rangle \;=\;\sum_{m=0}^{\infty }\sum_{k=0}^{\infty }M_{m+1}\left(t\right)\;M_{k+1}\left(t\right)\;\langle L^{m}\left(A\right)\;L^{k}\left(A^{†}\right)\rangle ,$$

where we use the linearity of the average value. Using m=n−k, Equation (112) takes the form

(113) $$\langle \Phi_{t}^{\left(M\right)}\left(A\right){\;\Phi }_{t}^{\left(M\right)}\left(A^{†}\right)\rangle \;=\;\sum_{m=0}^{\infty }\sum_{k=0}^{n}M_{n-k+1}\left(t\right)\;M_{k+1}\left(t\right)\;\langle L^{n-k}\left(A\right)\;L^{k}\left(A^{†}\right)\rangle .$$

Using (102) and (113), bi-positivity condition (104) is represented by the inequality

(114) $$\sum_{n=0}^{\infty }M_{n+1}\left(t\right)\;\langle L^{n}\left(A\;A^{†}\right)\rangle \;\geq \;\sum_{n=0}^{\infty }\sum_{k=0}^{n}M_{n-k+1}\left(t\right)\;M_{k+1}\left(t\right)\;\langle L^{n-k}\left(A\right)\;L^{k}\left(A^{†}\right)\rangle .$$

Since inequality (114) must hold for all t>0, we will look for conditions on L by using the inequalities

(115) $$M_{n+1}\left(t\right)\;\langle L^{n}\left(A\;A^{†}\right)\rangle \;\geq \;\sum_{k=0}^{n}M_{n-k+1}\left(t\right)\;M_{k+1}\left(t\right)\;\langle L^{n-k}\left(A\right)\;L^{k}\left(A^{†}\right)\rangle .$$

Using the assumption that the inequality

(116) $$M_{n}\left(t\right)\;>\;0$$

holds for all n∈ℕ and all t>0, we obtain the condition

(117) $$\langle L^{n}\left(A\;A^{†}\right)\rangle \;\geq \;\sum_{k=0}^{n}E_{k}^{n}\left(K,M\right)\;\langle L^{n-k}\left(A\right)\;L^{k}\left(A^{†}\right)\rangle ,$$

where

(118) $$E_{k}^{n}\left(K,M\right)\;:=\frac{M_{n-k+1}\left(t\right)\;M_{k+1}\left(t\right)}{M_{n+1}\left(t\right)}.$$

and $k,n\in {\mathbb{N}}_{0}$ and 0≤k≤n. □

**Definition** **9.** *Let a pair of kernels $\left(M\left(t\right),\;K\left(t\right)\right)$ belong to the Sonin set $S_{-1}$, and*

(119) $$M_{n}\left(t\right)\;>\;0\;\;$$

*for all*n∈ℕ*and all*t>0. *Then the function*

(120) $$E_{k}^{n}\left(K,M\right)\;:=\frac{M_{n-k+1}\left(t\right)\;M_{k+1}\left(t\right)}{M_{n+1}\left(t\right)},\;$$

*where*$k,\;n\in {\mathbb{N}}_{0}$*and*0≤k≤n*, will be called the general binomial coefficients.*

**Remark** **2.** *If the kernel $M\left(t\right)$ is positive*

(121) $$M\left(t\right)\;>\;0\;$$

*for all t>0, then condition (119) holds.*

**Definition** **10.** *Let the real operator L satisfy the inequalities*

$$\langle L^{n}\left({A\;A}^{†}\right)\rangle \;\geq \sum_{k=0}^{n}{\;E}_{k}^{n}\left(K,M\right)\;\langle L^{n-k}\left(A\right){\;L}^{k}\left(A^{†}\right)\rangle$$

*for all* $n\in {\mathbb{N}}_{0}$ *and all* A∈A *. Then* L *will be called the general dissipative superoperator.*

*The general complete dissipativity condition is defined in the form*

$$\langle L^{n}\left(A_{i}\;\;,A_{j}^{†}\right)\rangle \;\geq \;\sum_{k=0}^{n}E_{k}^{n}\left(K,M\right)\;\langle L^{n-k}\left(A_{i}\right)\;L^{k}\left(A_{j}^{†}\right)\rangle$$

*which holds for all t>0, and for all*$A_{i},A_{j}\in A$, j=1,…m*and all n,m∈ℕ.*

We will consider the bi-positivity condition instead of the complete positivity condition to simplify the description and proof in the next sections. Conditions for complete positivity were proved similarly to the proofs for bi-positivity. The condition for general complete dissipativity of the real superoperator L will be used analogously as the condition for general dissipativity.

In connection with general dissipativity condition (121), which must be satisfied for any value of all n∈ℕ, two questions arise: (1) What condition must the superoperator $L^{1}=L$ satisfy in order for the general dissipativity condition (121) to hold for all n∈ℕ? (2) What is the connection between the general dissipativity condition, and the condition of the dissipativity? Answers to these questions will be offered in the following sections.

#### 4.3.2. General Dissipativity for n=1

Let us consider the general dissipativity condition for the superoperator $L^{n}$ with n=1.

**Theorem** **6.** *Let a pair of kernels $\left(M\left(t\right),\;K\left(t\right)\right)$ belongs to the Sonin set $S_{-1}$, and*

$$M_{n}\left(t\right)\;>\;0\;$$

*for all n∈ℕ and all*t>0.

*Then the general binomial coefficients with* k=0 *and* k=n *are equal to one*

$$E_{0}^{n}\left(K,M\right)\;=E_{n}^{n}\left(K,M\right)\;=1$$

*for all n∈ℕ.*

**Proof.**  Using the definition of the general binomial coefficients, we have

(122) $$E_{0}^{n}\left(K,M\right)\;:=\frac{M_{n+1}\left(t\right)\;M_{1}\left(t\right)}{M_{n+1}\left(t\right)},$$

(123) $$E_{n}^{n}\left(K,M\right)\;:=\frac{M_{1}\left(t\right)\;M_{n+1}\left(t\right)}{M_{n+1}\left(t\right)}.$$

Using

(124) $$M_{1}\left(t\right)\;:=\;\left(K\;*\;M\right)\left(t\right)\;=\;\left\{1\right\},$$

we get

(125) $$E_{0}^{n}\left(K,M\right)\;=E_{n}^{n}\left(K,M\right)\;=\frac{\left\{1\right\}\;M_{n+1}\left(t\right)\;}{M_{n+1}\left(t\right)}\;=\left\{1\right\}.$$

 □

**Theorem** **7.** *Let a pair of kernels $\left(M\left(t\right),\;K\left(t\right)\right)$ belongs to the Sonin set $S_{-1}$, and*

$$M_{n}\left(t\right)\;>\;0\;$$

*for all n∈ℕ and all*t>0.

*Then the general dissipative superoperator* $L^{n}$ *with* n=1 *satisfies the condition*

(126) $$\langle L\left(A\;A^{†}\right)\rangle \;\geq \;\langle L\left(A\right)\;A^{†}+A\;L\left(A^{†}\right)\rangle$$

*for all A∈A.*

**Proof.**  For n=1, inequality (110) takes the form

(127) $$\langle L\left(A,A^{†}\right))\rangle \;\geq \;\langle E_{0}^{1}\left(K,M\right)\;L\left(A\right)\;A^{†}+E_{1}^{1}\left(K,M\right)\;A\;L\left(A^{†}\right)\rangle ,$$

where

(128) $$E_{0}^{1}\left(K,M\right)\;:=\frac{M_{2}\left(t\right)\;M_{1}\left(t\right)}{M_{2}\left(t\right)},$$

(129) $$E_{1}^{1}\left(K,M\right)\;:=\frac{M_{1}\left(t\right)\;M_{2}\left(t\right)}{M_{2}\left(t\right)}.$$

Using Theorem 7, we have

(130) $$E_{0}^{1}\left(K,M\right)\;=E_{1}^{1}\left(K,M\right)\;=\frac{\left\{1\right\}\;M_{2}\left(t\right)}{M_{2}\left(t\right)}\;=\;\left\{1\right\}.$$

Therefore, inequality (127) has the form

(131) $$\langle L\left(A,A^{†}\right)\rangle \;\geq \;\langle L\left(A\right)\;A^{†}+A\;L\left(A^{†}\right)\rangle .$$

**Theorem** **8.** *Let $\Phi_{t}^{\left(M\right)}$ be general non-Markovian map with the positive kernel*$M\left(t\right)\;>\;0$*for all*t>0*such that the bi-positivity condition*

(132) $$\langle \Phi_{t}^{\left(M\right)}\left(A\;A^{†}\right)\rangle \;\geq \;\langle \Phi_{t}^{\left(M\right)}\left(A\right)\;\Phi_{t}^{\left(M\right)}\left(A^{†}\right)\rangle \;$$

*is satisfied for all t>0, and all*A∈A.

*Then, the real superoperator L satisfies the dissipativity condition*

(133) $$\langle L\left(A\;A^{†}\right)\rangle \;\geq \;\langle L\left(A\right)\;A^{†}+A\;L\left(A^{†}\right)\rangle q$$

*holds for all A∈A.*

**Proof.**  Using Theorem 5, we get that the bi-positivity condition (132) for general non-Markovian map $\Phi_{t}^{\left(M\right)}$ leads to the general dissipativity condition for the superoperator $L^{n}$. □

Then, using Theorem 6, the general dissipativity condition leads to the dissipativity condition (133).

#### 4.3.3. From General Dissipativity to Bi-Positivity

Let us prove the theorem that is converse to Theorem 5.

**Theorem** **9.** *Let L be real superoperator, for which the inequalities*

(134) $$\langle L^{n}\left({A\;A}^{†}\right)\rangle \;\geq \sum_{k=0}^{n}{\;E}_{k}^{n}\left(K,M\right)\;\langle L^{n-k}\left(A\right){\;L}^{k}\left(A^{†}\right)\rangle \;$$

*are satisfied for all n∈ℕ, and all A∈A, where the functions $M_{n+1}\left(t\right)$ are positive*

(135) $$M_{n+1}\left(t\right)\;:=\;\left(K\;*\;M^{*,n+1}\right)\left(t\right)\;=\;\left(\left\{1\right\}\;*\;M^{*,n}\right)\left(t\right)\;>\;0$$

*for all $n\in {\mathbb{N}}_{0}$.*

*Then the bi-positivity condition*

(136) $$\langle \Phi_{t}^{\left(M\right)}\left(A\;A^{†}\right)\rangle \;\geq \;\langle \Phi_{t}^{\left(M\right)}\left(A\right)\;\Phi_{t}^{\left(M\right)}\left(A^{†}\right)\rangle \;$$

*is satisfied for all t>0 and all A∈A for the general non-Markovian map*

(137) $$\Phi_{t}^{\left(M\right)}\left(A\right)\;=\;\sum_{n=0}^{\infty }M_{n+1}\left(t\right){\;L}^{n}.$$

**Proof.**  Let us use condition (134) in the form

(138) $$\langle L^{n}\left(A\;A^{†}\right)\rangle \;\geq \;\sum_{k=0}^{n}\frac{M_{n-k+1}\left(t\right)\;M_{k+1}\left(t\right)}{M_{n+1}\left(t\right)}\;\langle L^{n-k}\left(A\right)\;L^{k}\left(A^{†}\right)\rangle .$$

Using assumption that $M_{n}\left(t\right)\;>\;0$ holds for all n∈ℕ and all t > 0, inequality (138) can be written as

(139) $$M_{n+1}\left(t\right)\;\langle L^{n}\left(A\;A^{†}\right)\rangle \;\geq \;\sum_{k=0}^{n}\left(M_{n-k+1}\left(t\right)\;M_{k+1}\left(t\right)\right)\;\langle L^{n-k}\left(A\right)\;L^{k}\left(A^{†}\right)\rangle .$$

Since, by condition of the theorem, inequality (138) holds for any $n\in {\mathbb{N}}_{0}$. Then, summing from 0 to ∞ inequality (139), we obtain

(140) $$\sum_{n=0}^{\infty }M_{n+1}\left(t\right)\;\langle L^{n}\left(A\;A^{†}\right)\rangle \;\geq \;\sum_{n=0}^{\infty }\sum_{k=0}^{n}\left(M_{n-k+1}\left(t\right)\;M_{k+1}\left(t\right)\right)\;\langle L^{n-k}\left(A\right)\;L^{k}\left(A^{†}\right)\rangle .$$

Using m=n−k, condition (140) takes the form

(141) $$\sum_{n=0}^{\infty }M_{n+1}\left(t\right)\;\langle L^{n}\left(A\;A^{†}\right)\rangle \;\geq \;\sum_{m=0}^{\infty }\sum_{k=0}^{\infty }M_{m+1}\left(t\right)\;M_{k+1}\left(t\right)\;\langle L^{m}\left(A\right)\;L^{k}\left(A^{†}\right)\rangle .$$

Then

(142) $$\sum_{n=0}^{\infty }M_{n+1}\left(t\right)\;\langle L^{n}\left(A\;A^{†}\right)\rangle \;\geq \;\left(\sum_{m=0}^{\infty }\sum_{k=0}^{\infty }\;M_{m+1}\left(t\right)\;M_{k+1}\left(t\right)\right)\;\langle L^{m}\left(A\right)\;L^{k}\left(A^{†}\right)\rangle .$$

(143) $$\langle \sum_{n=0}^{\infty }M_{n+1}\left(t\right)\;L^{n}\left(A\;A^{†}\right)\rangle \;\geq \;\langle \left(\sum_{m=0}^{\infty }M_{m+1}\left(t\right)\;L^{m}\left(A\right)\right)\;\left(\;\sum_{k=0}^{\infty }M_{k+1}\left(t\right)\;L^{k}\left(A^{†}\right)\right)\rangle .$$

(144) $$\langle \Phi_{t}^{\left(M\right)}\left(A\;A^{†}\right)\rangle \;\geq \;\langle \Phi_{t}^{\left(M\right)}\left(A\right){\;\Phi }_{t}^{\left(M\right)}\left(A^{†}\right)\rangle .$$

 □

As a result, Theorems 5 and 9 allow us to formulate the following statement.

**Corollary** **1.** *A necessary and sufficient condition for the bi-positivity property of the general non-Markovian quantum dynamical map $\Phi_{t}^{\left(M\right)}$ with positive kernel $M\left(t\right)$ in the form*

(145) $$\langle \Phi_{t}^{\left(M\right)}\left(A\;A^{†}\right)\rangle \;\geq \;\langle \Phi_{t}^{\left(M\right)}\left(A\right)\;\Phi_{t}^{\left(M\right)}\left(A^{†}\right)\rangle \;$$

*that holds for all t>0, is the general dissipativity of the real superoperator L in the form*

(146) $$\langle L^{n}\left({A\;A}^{†}\right)\rangle \;\geq \sum_{k=0}^{n}{\;E}_{k}^{n}\left(K,M\right)\;\langle L^{n-k}\left(A\right){\;L}^{k}\left(A^{†}\right)\rangle \;$$

*that satisfies for all n∈ℕ.*

**Remark** **3.** *Let the inequality*

(147) $$\langle L^{n}\left(A_{i}{\;A}_{j}^{†}\right)\rangle \;\geq \sum_{k=0}^{n}{\;E}_{k}^{n}\left(K,M\right)\;\langle L^{n-k}\left(A_{i}\right){\;L}^{k}\left(A_{j}^{†}\right)\rangle .$$

*be satisfied for all*n∈ℕ*and all*$A_{i},A_{j}\in A$*, where*i,j=1,…,m.

*Then, the completely positive condition holds in the form*

$$\sum_{i=1}^{m}\sum_{i=1}^{m}B_{i}^{†}\Phi_{t}^{\left(M\right)}\left(A_{i}^{†}\;A_{j}\right)B_{j}\geq 0.$$

*for all n∈ℕ and all $A_{i},B_{i}\in A$, where i,j=1,…,m.*

*The proof of this statement is realized similarly to Theorem 9.*

**Corollary** **2.** *The condition that a superoperator is dissipative is a necessary condition for non-Markovian maps to be bi-positive, but it is not a sufficient condition.*

#### 4.3.4. From General Dissipativity to Dissipativity

Let us consider the relationship between the concepts of dissipativity and general dissipativity.

**Theorem** **10.** *Let real superoperator L satisfy the general dissipativity condition*

(148) $$\langle L^{n}\left({A\;A}^{†}\right)\rangle \;\geq \sum_{k=0}^{n}{\;E}_{k}^{n}\left(K,M\right)\;\langle L^{n-k}\left(A\right){\;L}^{k}\left(A^{†}\right)\rangle$$

*for all n∈ℕ and all*A∈A.

*Then, the superoperator* L *satisfies the dissipativity condition*

(149) $$\langle L^{n}\left(A\;A^{†}\right)\rangle \;\geq \sum_{k=0}^{n}\;\left(\begin{array}{l} n\\ k \end{array}\right)\;\langle L^{n-k}\left(A\right)\;L^{k}\left(A^{†}\right)\rangle$$

*for all n∈ℕ.*

**Proof.**  Using Theorem 7, we get that general dissipativity condition (148) with n=1 has the form

(150) $$\langle L\left(A\;A^{†}\right)\rangle \;\geq \;\langle L\left(A\right)\;A^{†}+A\;L\left(A^{†}\right)\rangle .$$

Using inequality (150) two times for the expression $L^{2}\left(A\;A^{†}\right)$ and the linearity property of the superoperator L, we obtain

$$\langle L^{2}\left(A\;A^{†}\right)\rangle =\langle L\left(L\left(A\;A^{†}\right)\right)\rangle \;\geq \;\langle L\left(L\left(A\right)\;A^{†}+A\;L\left(A^{†}\right)\right)\rangle =\;\langle L\left(L\left(A\right)\;A^{†}\right)\;+\;L\left(A\;L\left(A^{†}\right)\right)\rangle \;\geq \;\;\langle L^{2}\left(A\right)\;A^{†}+2\;L\left(A\right)\;L\left(A^{†}\right)\;+\;A\;L^{2}\left(A^{†}\right)\rangle .$$

Using inequality (150) n-times for the expression $L^{n}\left(A\;A^{†}\right)$ and the linearity property of the superoperator L in a similar way, we obtain inequality (149). □

**Corollary** **3.** *Let the general non-Markovian map $\Phi_{t}^{\left(M\right)}$ with positive kernel $M\left(t\right)$ satisfy the bi-positivity condition in the form of the inequality*

(151) $$\langle \Phi_{t}^{\left(M\right)}\left(A\;A^{†}\right)\rangle \;\geq \;\langle \Phi_{t}^{\left(M\right)}\left(A\right)\;\Phi_{t}^{\left(M\right)}\left(A^{†}\right)\rangle ,\;$$

*which holds for all t>0 and all*A∈A.

*Then, the superoperator*L*satisfies the dissipativity condition*

(152) $$\langle L^{n}\left(A\;A^{†}\right)\rangle \;\geq \;\sum_{k=0}^{n}\left(\begin{array}{l} n\\ k \end{array}\right)\;\langle L^{n-k}\left(A\right)\;L^{k}\left(A^{†}\right)\rangle$$

*for all n∈ℕ, and all*A∈A.

**Remark** **4.** *The dissipativity condition*

(153) $$\langle L^{n}\left({A\;A}^{†}\right)\rangle \;\geq \;\sum_{k=0}^{n}\left(\begin{array}{l} n\\ k \end{array}\right)\;\langle L^{n-k}\left(A\right){\;L}^{k}\left(A^{†}\right)\rangle$$

*which holds for all n∈ℕ does not lead to the fact that the general non-Markov map $\Phi_{t}^{\left(M\right)}$ is bi-positive.*

*If we assume in non-Markovian quantum theory that* L *is dissipative superoperators only, then the standard bi-positive condition*

$$\Phi_{\tau }\left(A\;A^{†}\right)\;-\Phi_{\tau }\left(A\right)\;\Phi_{\tau }\left(A^{†}\right)\;\geq \;0.$$

*is replaced by the inequality*

$$\int_{0}^{t}\;d\tau \;\left(\Phi_{\tau }^{\left(M\right)}\left(A\;A^{†}\right)\;-\;\Phi_{t-\tau }^{\left(M\right)}\left(A\right)\;\Phi_{\tau }^{\left(M\right)}\left(A^{†}\right)\right)\;\geq \;0.$$

*that should be satisfied for all t≥0. Let us prove this statement.*

**Theorem** **11.** *Let real superoperator L satisfy the inequality*

(154) $$\langle L\left(A\;A^{†}\right)\rangle \;\geq \;\langle L\left(A\right)\;A^{†}+A\;L\left(A^{†}\right)\rangle .\;$$

*Then, the inequality*

$$\int_{0}^{t}\langle \Phi_{\tau }^{\left(M\right)}\left(A\;A^{†}\right)\rangle \;d\tau \;\geq \;\int_{0}^{t}\langle \Phi_{t-\tau }^{\left(M\right)}\left(A\right)\;\Phi_{\tau }^{\left(M\right)}\left(A^{†}\right)\rangle \;d\tau$$

*holds for all t>0 and all A∈A, where $\Phi_{\tau }^{\left(M\right)}$ is general non-Markovian map.*

**Proof.**  Using the proof of Theorem 10, we get that the repeated action of the inequality (154) gives the condition

$$\langle L^{n}\left(A\;A^{†}\right)\rangle \;\geq \;\sum_{k=0}^{n}\left(\begin{array}{l} n\\ k \end{array}\right)\;\langle L^{n-k}\left(A\right)\;L^{k}\left(A^{†}\right)\rangle .$$

Using the series representation of $\Phi_{t}\left(A\;A^{†}\right)$ and inequality (18), we get

$$\langle \Phi_{t}^{\left(M\right)}\left(A\;A^{†}\right)\rangle \;=\;\sum_{k=0}^{\infty }M_{n+1}\left(t\right)\;\langle L^{n}\left(A\;A^{†}\right)\rangle \;\geq \;\;\sum_{n=0}^{\infty }\sum_{k=0}^{n}\;M_{n+1}\left(t\right)\;\left(\begin{array}{l} n\\ k \end{array}\right)\;\langle L^{n-k}\left(A\right)\;L^{k}\left(A^{†}\right)\rangle \;=\;\;\sum_{m=0}^{\infty }\sum_{k=0}^{\infty }\;M_{m+k+1}\left(t\right)\;\left(\begin{array}{l} m+k\\ k \end{array}\right)\;\langle L^{m}\left(A\right)\;L^{k}\left(A^{†}\right)\rangle .$$

Using

$$\left(\begin{array}{l} m+k\\ k \end{array}\right)\;\geq \;1$$

for $k,n\in {\mathbb{N}}_{0}$, 0≤k≤n, and $M_{n+1}\left(t\right)\;>\;0$ for all $n\in {\mathbb{N}}_{0}$, we can write the inequality

$$\langle \Phi_{t}^{\left(M\right)}\left(A\;A^{†}\right)\rangle \;\geq \;\sum_{m=0}^{\infty }\sum_{k=0}^{\infty }M_{m+k+1}\left(t\right)\;\langle L^{m}\left(A\right)\;L^{k}\left(A^{†}\right)\rangle .$$

The convolution with $\left\{1\right\}$ means the integration in the form

$$\int_{0}^{t}f\left(\tau \right)\;d\tau \;=\;\left(\left\{1\right\}\;*\;f\right)\left(t\right),$$

Therefore, multiplying the left and right sides of the inequality by $\left\{1\right\}$ and using Sonin’s condition

$$\left(K\;*\;M\right)\left(t\right)\;=\;\left\{1\right\},$$

We obtain

$$\left(\left\{1\right\}\;*\;\langle \Phi_{t}^{\left(M\right)}\left(A\;A^{†}\right)\rangle \right)\left(t\right)\;\geq \;\left(\left\{1\right\}\;*\;\sum_{m=0}^{\infty }\sum_{k=0}^{\infty }M_{m+k+1}\left(t\right)\;\langle L^{m}\left(A\right)\;L^{k}\left(A^{†}\right)\rangle \right)\left(t\right)\;=\;\;\sum_{m=0}^{\infty }\sum_{k=0}^{\infty }\;\left(\left\{1\right\}\;*\;M^{*,m}\;*\;\left\{1\right\}\;*\;M^{*,k}\right)\left(t\right)\;\langle L^{m}\left(A\right)\;L^{k}\left(A^{†}\right)\rangle \;=\;\;\langle \left(\left(\sum_{m=0}^{\infty }\;\left(\left\{1\right\}\;*\;M^{*,m}\right)\left(t\right)\;L^{m}\left(A\right)\right)\;*\;\left(\sum_{k=0}^{\infty }\;\left(\left\{1\right\}\;*\;M^{*,k}\right)\left(t\right)\;L^{k}\left(A^{†}\right)\right)\right)\rangle \;=\;\;\langle \left(\Phi_{t}^{\left(M\right)}\left(A\right)\;*\;\Phi_{t}^{\left(M\right)}\left(A^{†}\right)\right)\rangle \;=\;\int_{0}^{t}\langle \Phi_{t-\tau }^{\left(M\right)}\left(A\right)\Phi_{\tau }^{\left(M\right)}\left(A^{†}\right)\rangle \;d\tau .$$

 □

**Remark** **5.** *Alternative forms of writing this inequality*

$$\int_{0}^{t}\langle \Phi_{\tau }^{\left(M\right)}\left(A\;A^{†}\right)\;-{\;\Phi }_{t-\tau }^{\left(M\right)}\left(A\right)\Phi_{\tau }^{\left(M\right)}\left(A^{†}\right)\rangle \;d\tau \;\geq \;0,\;\langle \Phi_{t}^{\left(M\right)}\left(A\;A^{†}\right)\rangle \;\geq \;\frac{d}{dt}\int_{0}^{t}\langle \Phi_{t-\tau }^{\left(M\right)}\left(A\right)\;\Phi_{\tau }^{\left(M\right)}\left(A^{†}\right)\rangle \;d\tau .$$

**Remark** **6.** *Similarly, we can prove the inequality*

$$\int_{0}^{t}d\tau \langle \sum_{k=1}^{n}\sum_{l=1}^{n}B_{k}^{†}\Phi_{t}^{\left(M\right)}\left(A_{k}^{†}\;A_{l}\right)B_{l}\rangle \;\geq \;\int_{0}^{t}d\tau {\langle \sum_{k=1}^{n}\Phi_{t-\tau }^{\left(M\right)}\left(A_{k}\right)B_{k})}^{†}\;(\sum_{l=1}^{n}\Phi_{\tau }^{\left(M\right)}\left(A_{l}\right)B_{l}\rangle ,$$

*where we use $\Phi_{t}^{\left(M\right)}\left(A^{†}\right)\;={\left(\Phi_{t}^{\left(M\right)}\left(A\right)\right)}^{†}$.*

#### 4.3.5. From Dissipativity to Bi-Positivity

Let us consider the relationship between the dissipativity and bi-positivity in general non-Markovian dynamics.

**Theorem** **12.** *Let real superoperator L satisfy the dissipativity condition*

(155) $$\langle L^{n}\left({A\;A}^{†}\right)\rangle \;\geq \;\sum_{k=0}^{n}\left(\begin{array}{l} n\\ k \end{array}\right)\;\langle L^{n-k}\left(A\right){\;L}^{k}\left(A^{†}\right)\rangle$$

*for all*n∈ℕ*and all A∈A, and the general binomial coefficients satisfy the condition*

(156) $$\left(\begin{array}{l} n\\ k \end{array}\right)\;\geq \;E_{k}^{n}\left(K,M\right)\;$$

*for all t>0 and all*$k,n\in {\mathbb{N}}_{0}$, 0≤k≤n.

*Then, the superoperator* L *satisfies the general dissipativity condition*

(157) $$\langle L^{n}\left({A\;A}^{†}\right)\rangle \;\geq \sum_{k=0}^{n}{\;E}_{k}^{n}\;\langle L^{n-k}\left(A\right){\;L}^{k}\left(A^{†}\right)\rangle$$

*for all* n∈ℕ *and all* A∈A.

*Then, the general non-Markovian map* $\Phi_{t}^{\left(M\right)}$ *satisfies the bi-positivity condition*

(158) $$\langle \Phi_{t}^{\left(M\right)}\left(A\;A^{†}\right)\rangle \;\geq \;\langle \Phi_{t}^{\left(M\right)}\left(A\right)\;\Phi_{t}^{\left(M\right)}\left(A^{†}\right)\rangle ,$$

*which holds for all t>0.*

**Proof.**  Using inequality (156), we get

(159) $$\sum_{k=0}^{n}\left(\begin{array}{l} n\\ k \end{array}\right)\;\langle L^{n-k}\left(A\right)\;L^{k}\left(A^{†}\right)\rangle \;\geq \;\sum_{k=0}^{n}E_{k}^{n}\left(K,M\right)\;\langle L^{n-k}\left(A\right)\;L^{k}\left(A^{†}\right)\rangle .$$

Then, using inequalities (155) and (159), we obtain the general dissipativity condition

(160) $$\langle L^{n}\left(A\;A^{†}\right)\rangle \;\geq \;\sum_{k=0}^{n}E_{k}^{n}\left(K,M\right)\;\langle L^{n-k}\left(A\right)\;L^{k}\left(A^{†}\right)\rangle$$

for all n∈ℕ.

Using Theorem 9, condition (160) gives the bi-positivity condition (158). □

**Theorem** **13.** *Let the general non-Markovian map $\Phi_{t}^{\left(M\right)}$ with positive kernel $M\left(t\right)$ satisfy the bi-positivity condition in the form of the inequality*

(161) $$\langle \Phi_{t}^{\left(M\right)}\left(A\;A^{†}\right)\rangle \;\geq \;\langle \Phi_{t}^{\left(M\right)}\left(A\right)\;\Phi_{t}^{\left(M\right)}\left(A^{†}\right)\rangle ,\;$$

*which holds for all t>0 and all A∈A, and the general binomial coefficients satisfy the condition*

(162) $$E_{k}^{n}\left(K,M\right)\;\geq \;\left(\begin{array}{l} n\\ k \end{array}\right)\;$$

*for all t>0 and all*$k,n\in {\mathbb{N}}_{0}$, 0≤k≤n.

*Then, the superoperator*L*satisfies the dissipativity condition*

(163) $$\langle L^{n}\left(A\;A^{†}\right)\rangle \;\geq \;\sum_{k=0}^{n}\left(\begin{array}{l} n\\ k \end{array}\right)\;\langle L^{n-k}\left(A\right)\;L^{k}\left(A^{†}\right)\rangle$$

*for all*n∈ℕ*and all*A∈A.

**Proof.**  Using Theorem 5, bi-positivity condition (161) gives the general dissipativity condition

(164) $$\langle L^{n}\left(A\;A^{†}\right)\rangle \;\geq \;\sum_{k=0}^{n}E_{k}^{n}\left(K,M\right)\;\langle L^{n-k}\left(A\right)\;L^{k}\left(A^{†}\right)\rangle$$

for all n∈ℕ and all A∈A. Then using the condition (162) for the general binomial coefficients, we obtain

(165) $$\sum_{k=0}^{n}E_{k}^{n}\left(K,M\right)\;\langle L^{n-k}\left(A\right)\;L^{k}\left(A^{†}\right)\rangle \;\geq \;\sum_{k=0}^{n}\left(\begin{array}{l} n\\ k \end{array}\right)\;\langle L^{n-k}\left(A\right)\;L^{k}\left(A^{†}\right)\rangle$$

for all n∈ℕ and all A∈A. Then, using conditions (164) and (165), we derive the dissipativity condition (163).

#### 4.3.6. Examples of General Binomial Coefficients

Let us consider the general binomial coefficients for the non-Markovian quantum maps with the pair of kernels

(166) $$M\left(t\right)\;=h_{\alpha }\left(t\right)\;=\frac{t^{\alpha -1}}{\Gamma \left(\alpha \right)},\;K\left(t\right)\;=h_{1-\alpha }\left(t\right)\;=\frac{t^{-\alpha }}{\Gamma \left(1-\alpha \right)}$$

that belongs to the Sonin set for $\alpha \in \;\left(0,1\right)$. We see that the condition $M\left(t\right)\;>0$ holds for all t>0.

Let us give a definition of the generalized binomial coefficients (see [26] (p. 14–15) and [29] (p. 26–27)). These well-known “generalized” coefficients should not be confused with the “general” coefficients suggested in this article.

**Definition** **11.** *The generalized binomial coefficients are defined by the equation*

(167) $$\left(\begin{array}{l} \alpha \\ \beta \end{array}\right)\;:=\frac{\Gamma \left(\alpha +1\right)}{\Gamma \left(\beta +1\right)\Gamma \left(\alpha -\beta +1\right)},\;$$

*where*α,β∈ℂ, α≠−1,−2,…

Let us prove that the general binomial coefficients for kernel pair (166) are expressed through the generalized binomial coefficients.

**Theorem** **14.** *Let the pair of kernels $\left(M\left(t\right),\;K\left(t\right)\right)$ be defined by Equation (166).*

*Then, we have the equation*

$$M_{n+1}\left(t\right)\;=\;h_{\alpha n+1}\left(t\right),\;$$

*which satisfies the condition $M_{n}\left(t\right)\;>0$ for all n∈ℕ and all t>0.*

*The general binomial coefficients are defined by the equation*

(168) $$E_{k}^{n}\left(K,M\right)\;=\;E_{k}^{n}\left(h_{1-\alpha },h_{\alpha }\right)\;=\;\left(\begin{array}{l} \alpha n\\ \alpha k \end{array}\right),\;$$

*where $\alpha \in \;\left(0,1\right)$, $k,n\in {\mathbb{N}}_{0}$, 0≤k≤n.*

**Proof.**  Using the property

$$\left(h_{\alpha }\;*\;h_{\beta }\right)\left(t\right)\;=h_{\alpha +\beta }\left(t\right)$$

that holds for α,β>0, we obtain

$$M^{*,n}\left(t\right)\;={\left(h_{\alpha }\right)}^{*,n}\;=h_{\alpha n}\left(t\right).$$

Then,

(169) $$M_{n+1}\left(t\right)\;=\;\left(K\;*\;M^{*,n+1}\right)\left(t\right)\;=\;\left(h_{1-\alpha }\;*\;h_{\alpha \left(n+1\right)}\right)\left(t\right)\;=\;\frac{t^{\alpha n}}{\Gamma \left(\alpha n+1\right)}\;=h_{\alpha \left(n+1\right)+1-\alpha }\left(t\right)\;=h_{\alpha n+1}\left(t\right).$$

We see that the inequality $M_{n+1}\left(t\right)\;>\;0$ holds for all n∈ℕ and for all t>0.

Then, using (169), we get

(170) $$M_{n-k+1}\left(t\right)\;M_{k+1}\left(t\right)\;=h_{\alpha \left(n-k\right)+1}\left(t\right)\;h_{\alpha k+1}\left(t\right)\;=\;\frac{t^{\alpha \left(n-k\right)}}{\Gamma \left(\alpha \left(n-k\right)\;+\;1\right)}\;\frac{t^{\alpha k}}{\Gamma \left(\alpha k+1\right)}\;=\;\frac{\Gamma \left(\alpha n+1\right)}{\Gamma \left(\alpha k+1\right)\;\Gamma \left(\alpha \left(n-k\right)\;+\;1\right)}\;\frac{t^{\alpha n}}{\Gamma \left(\alpha n+1\right)}\;=\;\left(\begin{array}{l} \alpha n\\ \alpha k \end{array}\right)\;h_{\alpha n+1}\left(t\right),$$

where the generalized binomial coefficients $\left(\begin{array}{l} \alpha n\\ \alpha k \end{array}\right)$ are defined by (167).

As a result, using (170), we derive the equation

(171) $$E_{k}^{n}\left(K,M\right)\;=E_{k}^{n}\left(h_{1-\alpha },h_{\alpha }\right)\;=\frac{M_{n-k+1}\left(t\right)\;M_{k+1}\left(t\right)}{M_{n+1}\left(t\right)}\;=\;\left(\begin{array}{l} \alpha n\\ \alpha k \end{array}\right),$$

where $\alpha \in \;\left(0,1\right)$, $k,n\in {\mathbb{N}}_{0}$, 0≤k≤n. □

Let us prove the following statement about the properties of the general binomial coefficients (171) for the kernels (166).

**Theorem** **15.** *Let us consider the function*

$$f_{n,k}\left(\alpha \right)\;:=\;\left(\begin{array}{l} \alpha n\\ \alpha k \end{array}\right)\;=\frac{\Gamma \left(\alpha n+1\right)}{\Gamma \left(\alpha k+1\right)\Gamma \left(\alpha \left(n-k\right)\;+\;1\right)},\;$$

*where α>0, 0≤k≤n, $k,n\in {\mathbb{N}}_{0}$. This function is increasing with respect to α>0 with fixed parameters $k,n\in {\mathbb{N}}_{0}$, and the following inequality holds*

$$f_{n,k}\left(\beta \right)\;\geq \;f_{n,k}\left(1\right)\;\geq \;f_{n,k}\left(\alpha \right),\;$$

*if*β≥1≥α>0.

**Proof.**  Let us prove that

$$\frac{d}{d\alpha }f_{n,k}\left(\alpha \right)\;\geq \;0.$$

Using the equation

$$\frac{d}{d\alpha }\Gamma \left(\alpha \right)\;=\;\Gamma \left(\alpha \right)\;\psi \left(\alpha \right),$$

where $\psi \left(\alpha \right)$ is the digamma function of real argument α>0, we obtain

(172) $$\frac{d}{d\alpha }f_{n,k}\left(\alpha \right)\;=f_{n,k}\left(\alpha \right)\;\left(n\;\psi \left(n\alpha +1\right)\;-\;k\;\psi \left(k\alpha +1\right)\;-\;\left(n-k\right)\;\psi \left(\left(n-k\right)\alpha +1\right)\right).$$

Using n=m+k, Equation (172) can be written in the form

$$\frac{d}{d\alpha }f_{n,k}\left(\alpha \right)\;=f_{m+k,k}\left(\alpha \right)\;(m\;\left(\psi \left(\left(m+k\right)\alpha +1\right)\;-\;\psi \left(m\alpha +1\right)\right)\;+\;\;k\;\left(\psi \left(\left(m+k\right)\alpha +1\right)\;-\;\psi \left(k\alpha +1\right)\right)).$$

Using the digamma function $\psi \left(\alpha \right)$ of real argument α>0, increases function with respect to α>0:

$$\psi \left(\left(m+k\right)\alpha +1\right)\;-\;\psi \left(m\alpha +1\right)\;\geq \;0$$

for k≥0, and that the generalized binomial coefficients are positive functions

$$f_{n,k}\left(\alpha \right)\;:=\;\left(\begin{array}{l} \alpha n\\ \alpha k \end{array}\right)\;>\;0$$

for α>0, 0≤k≤n, $k,n\in {\mathbb{N}}_{0}$, we derive that the generalized binomial coefficients $f_{n,k}\left(\alpha \right)$ as a function of the variable α>0 has a non-negative derivative $df_{n,k}\left(\alpha \right)/d\alpha \;\geq \;0$ with respect to α. □

**Remark** **7.** *For general binomial coefficients*

$$E_{k}^{n}\left(K,M\right)\;=\;\left(\begin{array}{l} \alpha n\\ \alpha k \end{array}\right),\;$$

*we have the inequalities*

$$\left(\begin{array}{l} n\\ k \end{array}\right)\;\geq \;\left(\begin{array}{l} \alpha n\\ \alpha k \end{array}\right),\;\;\;\;(0<\alpha <1),\;\left(\begin{array}{l} \alpha n\\ \alpha k \end{array}\right)\;\geq \;\left(\begin{array}{l} n\\ k \end{array}\right),\;\;\;\;(\alpha >1).\;$$

#### 4.3.7. Examples of Inequalities for General Binomial Coefficients

Let us give some examples of inequalities for general binomial coefficients.

**Example** **1.** *Let us consider the pair of the kernels*

(173) $$M\left(t\right)\;=h_{\alpha }\left(t\right)\;=\frac{t^{\alpha -1}}{\Gamma \left(\alpha \right)},\;\;\;\;K\left(t\right)\;=h_{1-\alpha }\left(t\right)\;=\frac{t^{-\alpha }}{\Gamma \left(1-\alpha \right)}.\;$$

*This pair belongs to the Sonin set* $S_{-1}$ *, if* $\alpha \in \;\left(0,1\right)$ *. For these kernels, the general binomial coefficients are given by the equations*

$$E_{k}^{n}\left(K,M\right)\;=E_{k}^{n}\left(h_{1-\alpha },h_{\alpha }\right)\;=\;\left(\begin{array}{l} \alpha n\\ \alpha k \end{array}\right)\;=\frac{\Gamma \left(\alpha n+1\right)}{\Gamma \left(\alpha k+1\right)\;\Gamma \left(\alpha \left(n-k\right)\;+\;1\right)}.\;$$

*If the parameter α satisfies the condition* $\alpha \in \;\left(0,1\right)$ *, then the inequalities*

$$\left(\begin{array}{l} n\\ k \end{array}\right)\;\geq \;E_{k}^{n}\left(K,M\right)\;$$

*hold for all* $k,n\in {\mathbb{N}}_{0}$ *, when 0≤k≤n and all t>0.*

**Example** **2.** *If the general binomial coefficients are given by equations*

$$E_{k}^{n}\left(K,M\right)\;=\;\left(\begin{array}{l} \alpha n\\ \alpha k \end{array}\right)\;$$

*with $\alpha \in \;\left(1,2\right)$, then the inequalities*

$$E_{k}^{n}\left(K,M\right)\;\geq \;\left(\begin{array}{l} n\\ k \end{array}\right)\;$$

*hold for all $k,n\in {\mathbb{N}}_{0}$, when 0≤k≤n.*

Note that the expressions of the general non-Markovian map $\Phi_{t}^{\left(M\right)}$ and the general binomial coefficients $E_{k}^{n}\left(K,M\right)$ are derived for the kernels $\left(M\left(t\right),K\left(t\right)\right)$ that belongs to the Sonin set. Kernels (173) with $\alpha \in \;\left(1,2\right)$ do not belong to the Sonin set $S_{-1}$.

We can assume that within the framework of the GFC of arbitrary order [59,71], the expression of the general non-Markovian map $\Phi_{t}^{\left(M\right)}$ and the general binomial coefficients $E_{k}^{n}\left(K,M\right)$ can also be derived.

For the kernels

(174) $$M\left(t\right)\;=h_{\alpha }\left(t\right)\;=\frac{t^{\alpha -1}}{\Gamma \left(\alpha \right)},\;K\left(t\right)\;=h_{2-\alpha }\left(t\right)\;=\frac{t^{1-\alpha }}{\Gamma \left(2-\alpha \right)}.$$

with $\alpha \in \;\left(1,2\right)$, the non-Markovian equation for quantum observables has the form

(175) $$D_{h_{2-\alpha }}^{t,*}\left[\tau \right]A\left(\tau \right)\;=L\;A\left(t\right),$$

where $D_{h_{2-\alpha }}^{t}\left[\tau \right]$ is the Caputo fractional derivative of the order $\alpha \in \;\left(1,2\right)$ that is defined as

$$D_{h_{2-\alpha }}^{t}\left[\tau \right]A\left(\tau \right)\;:=I_{h_{\alpha }}^{t}\left[\tau \right]\;A^{\left(2\right)}\left(\tau \right).$$

To solve Equation (175) and derive general binomial coefficients, we can use Theorem 4.3 and Example 4.10 of [29] (p. 231–231). The solution is described by the equation

$$A\left(t\right)\;=A\left(0\right)\;E_{\alpha ,1}\left[t^{\alpha }L\right]\;+\;A^{\left(1\right)}\left(0\right)\;t\;E_{\alpha ,2}\left[t^{\alpha }L\right],$$

where $E_{\alpha ,\beta }\left[z\right]$ is the two-parameter Mittag–Leffler function

$$E_{\alpha ,\beta }\left[t^{\alpha }L\right]\;=\;\sum_{n=0}^{\infty }\frac{t^{\alpha n}}{\Gamma \left(\alpha n+\beta \right)}\;L^{n}$$

For $A\left(0\right)\;=A$ and $A^{\left(1\right)}\left(0\right)\;=0$, we have the non-Markovian map

(176) $$\Phi_{t}^{\left(M\right)}\left(A\right)\;=\;E_{\alpha ,1}\left[t^{\alpha }L\right]\;A\;=\;\sum_{n=0}^{\infty }h_{\alpha n+1}\;L^{n}\left(A\right).$$

**Corollary** **4.** *Let the general dissipativity condition*

(177) $$\langle L^{n}\left(A\;A^{†}\right)\rangle \;\geq \;\sum_{k=0}^{n}E_{k}^{n}\left(h_{2-\alpha },h_{\alpha }\right)\;\langle L^{n-k}\left(A\right)\;L^{k}(A^{†})\rangle$$

*with the kernels (174), be satisfied for all n∈ℕ and*A∈A.

*Then, the general binomial coefficients have the form*

(178) $$E_{k}^{n}\left(h_{2-\alpha },h_{\alpha }\right)\;=\;\left(\begin{array}{l} \alpha n\\ \alpha k \end{array}\right)\;\geq \;\;\left(\begin{array}{l} n\\ k \end{array}\right),\;$$

*where*$\alpha \in \;\left(1,2\right)$.

*For the non-Markovian map (176), the bi-positivity condition*

(179) $$\langle \Phi_{t}^{\left(M\right)}\left(A\;A^{†}\right)\rangle \;\geq \;\langle \Phi_{t}^{\left(M\right)}\left(A\right)\;\Phi_{t}^{\left(M\right)}\left(A^{†}\right)\rangle$$

*is satisfied for all t>0 and all*A∈A.

## 5. Non-Markovian Quantum Oscillator with Nonlocality in Time

Let us consider a non-Markovian quantum oscillator with nonlocality in time.

As is usually assumed for oscillators that are open quantum system [10,11,12,37], the general form of a bounded completely dissipative superoperator holds for an unbounded superoperator L. Then, the general non-Markovian dynamics of coordinate Q and momentum P is described by the equations

(180) $$D_{\left(K\right)}^{t,*}\left[\tau \right]Q\left(\tau \right)\;=\;LQ\left(t\right),$$

(181) $$D_{\left(K\right)}^{t,*}\left[\tau \right]Q\left(\tau \right)\;=\;LP\left(t\right),$$

where L is defined by Equation (20). For the linear quantum oscillator, the operators $V_{k}=V_{k}\left(Q,P\right)$ and $H=H\left(Q,P\right)$ are the functions of the coordinate and momentum operators in the form

(182) $$H=\frac{1}{2m}P^{2}+\frac{m\omega^{2}}{2}Q^{2}+\frac{\mu }{2}\left(QP+PQ\right),$$

(183) $$V_{k}=a_{k}\;P+b_{k}\;Q,$$

where $a_{k}$, and $b_{k}$, k=1,2, are complex numbers. The term with parameter μ can be interpreted as friction, for which force is proportional to the velocity $m^{-1}P$.

**Remark** **8.** *In general, the operators coordinate Q and momentum P are unbounded operators. Due to this, instead of the Hilbert space, one can use the so-called rigged Hilbert space (the Gelfand triplet). A rigged Hilbert space is the ordered triplet*

$$B\subset H=H^{*}\subset B^{*},\;$$

*where H is a Hilbert space, B is a Banach space, and $B^{*}$ is dual of B. The term “rigged Hilbert space” is also used to describe the dual pairs (B, $B^{*}$) generated from a Hilbert space H. The term “Gelfand triplet” is sometimes used instead of the term “rigged Hilbert space”. Example of a rigged Hilbert space is the triple of spaces that consists of the Banach space $J\left({\mathbb{R}}^{n}\right)$ of test functions, the Hilbert space $L^{2}\left({\mathbb{R}}^{n}\right)$ of square integrable functions, and the Banach space $J^{*}\left({\mathbb{R}}^{n}\right)$ of the linear functionals on $J\left({\mathbb{R}}^{n}\right)$. For details, see Chapter 2 in [5].*

**Remark** **9.** *The Lindblad result has been extended by E.B. Davies [9] to a class of quantum dynamical semi-group with unbounded generating superoperators.*

**Remark** **10.** *In this linear model, the parameters $a_{k},b_{k}\in \mathbb{C}$ cannot be arbitrary. Let us consider the real parameters*

$$d_{aa}\;:=\;\frac{\hbar }{2}\left(|a_{1}{|}^{2}\;+\;|a_{2}{|}^{2}\right),\;\;\;\;d_{bb}\;:=\;\frac{\hbar }{2}\left(|b_{1}{|}^{2}\;+\;|b_{2}{|}^{2}\right),\;\;\;\;\;$$

$$d_{ab}\;:=\;-\frac{\hbar }{2}Re\left(a_{1}^{*}b_{1}+a_{2}^{*}b_{2}\right),\;\;\;c_{ab}=\;\frac{\hbar }{2}\;Im\left(a_{1}b_{1}^{*}+a_{2}b_{2}^{*}\right).$$

*There is a fundamental constraint [12] on the parameters in the form*

$$d_{aa}\;d_{bb}-d_{ab}^{2}\;\geq \;c_{ab}^{2},\;$$

*which follows from the Schwartz inequality*

$${\left(Re\sum_{k}a_{k}^{*}\;b_{k}\right)}^{2}\;+\;{\left(Im\sum_{k}a_{k}^{*}\;b_{k}\right)}^{2}\leq \sum_{k}{\left|a_{k}\right|}^{2}\;\sum_{k}{\left|b_{l}\right|}^{2}.$$

Using the canonical commutation relations for operators Q and P, we obtain Equations (180) and (181) for operators $Q\left(t\right)$ and $P\left(t\right)$ in the form

(184) $$D_{\left(K\right)}^{t,*}\left[\tau \right]Q\left(\tau \right)\;=\frac{1}{m}P\left(t\right)\;+\;\left(\mu -\lambda \right)Q\left(t\right),$$

(185) $$D_{\left(K\right)}^{t,*}\left[\tau \right]P\left(\tau \right)\;=\;-m\omega^{2}Q\left(t\right)\;-\;\left(\mu +\lambda \right)P\left(t\right),$$

where

(186) $$\lambda =Im\left(a_{1}b_{1}^{*}+a_{2}b_{2}^{*}\right),$$

where $D_{\left(K\right)}^{t,*}$ is the general fractional derivative with the kernel $K\left(t\right)\;\in \;C_{1}\left(0,\infty \right)$, for which $M\left(t\right)$ is the associated kernel so that the pair $\left(M\left(t\right),K\left(t\right)\right)$ belongs to the Sonin set $S_{-1}$.

For the kernel $K\left(t\right)\;=h_{1-\alpha }\left(t\right)\;=t^{-\alpha }/\Gamma \left(1-\alpha \right)$ with 0<α≤1, Equations (184) and (185) describe the exactly solvable model of non-Markovian dynamics that was first proposed in [37,38,39]. The exact solutions of equation for this case are derived in these works. For α=1, this model gives the standard Markovian quantum model [8,10,11,12].

Let us represent Equations (184) and (185) in the matrix form. Using the matrices

(187) $$A\left(t\right)\;=\;\left(\begin{array}{l} Q\left(t\right)\\ P\left(t\right) \end{array}\right),\;N\;=\;\left(\begin{array}{ll} \mu -\lambda & m^{-1}\\ -m\omega^{2} & -\mu -\lambda \end{array}\right),$$

Equations (184) and (185) are written as

(188) $$D_{\left(K\right)}^{t,*}\left[\tau \right]A\left(\tau \right)\;=N\;A\left(t\right),$$

where we used $LA\left(t\right)\;=N\;A\left(t\right)$.

**Theorem** **16.** *Let the function $K\left(t\right),M\left(t\right)\;\in C_{1}\left(0,\infty \right)$ belong to the Sonin set $S_{-1}$. Then, the initial value problem for the equation*

(189) $$D_{\left(K\right)}^{t,*}\left[\tau \right]A\left(\tau \right)\;=N\;A\left(t\right),\;$$

*and the condition $A\left(0\right)\;=A_{0}$, *where*$A\left(t\right)$*and*N are defined by (187), has the solution in the form*

(190) $$A\left(t\right)\;=\Phi_{t}^{\left(M\right)}A_{0},$$

*with the quantum dynamical map*

(191) $$\Phi_{t}^{\left(M\right)}\;=\;L\left(M,N,t\right)\;=\;\sum_{j=1}^{\infty }\left({K\;*\;M}^{*,j}\right)\left(t\right){\;N}^{j-1}$$

*where $L\left(M,N,t\right)$ is the second Luchko function with the matrix argument*N.

The statement of Theorem 16 follows directly from Theorem 3.

**Example** **3.** *For the kernel $K\left(t\right)\;=h_{1-\alpha }\left(t\right)\;=t^{-\alpha }/\Gamma \left(1-\alpha \right)$ with 0<α≤1, the GFD is the Caputo fractional derivative of the order $\alpha \in \;\left(0,1\right)$, and solution (191) has the form*

(192) $$\Phi_{t}^{\left(h_{\alpha }\right)}\;={\;E}_{\alpha }\left[t^{\alpha }N\right]\;=\;\sum_{k=0}^{\infty }\frac{t^{k\alpha }}{\Gamma \left(\alpha k+1\right)}{\;N}^{n}.$$

*For α=1, solution (192) gives*

(193) $$\Phi_{t}\;={\;e}^{\mathrm{tN}}\;=\;\sum_{n=0}^{\infty }\frac{t^{n}}{n!}{\;N}^{n}$$

*that describes the Markovian quantum dynamics of open system without nonlocality in time.*

Let us prove the following theorem.

**Theorem** **17.** *Equations (184) and (185) that describe the general non-Markovian quantum dynamics for coordinate and momentum have the solutions*

(194) $$Q\left(t\right)\;=\;\left(Ch_{\left(M\right)}\left[\lambda ,\nu ,t\right]\;+\;\frac{\mu }{\nu }\;Sh_{\left(M\right)}\left[\lambda ,\nu ,t\right]\right)Q_{0}+\frac{1}{m\nu }\;Sh_{\left(M\right)}\left[\lambda ,\nu ,t\right]P_{0},\;$$

(195) $$P\left(t\right)\;=-\frac{m\omega^{2}}{\nu }\;Sh_{\left(M\right)}\left[\lambda ,\nu ,t\right]Q_{0}\;+\;\left(Ch_{\left(M\right)}\left[\lambda ,\nu ,t\right]\;-\;\frac{\mu }{\nu }\;Sh_{\left(M\right)}\left[\lambda ,\nu ,t\right]\right)P_{0},$$

*where the functions $Sh_{\left(M\right)}\left[\lambda ,\nu ,t\right]$ and $Ch_{\left(M\right)}\left[\lambda ,\nu ,t\right]$ are defined by the expressions*

(196) $$Sh_{\left(M\right)}\left[\lambda ,\nu ,t\right]\;=\frac{1}{2}\left(L\left(M,\left(-\lambda +\nu \right),t\right)\;-\;L\left(M,\left(-\lambda -\nu \right),t\right)\right),\;$$

(197) $$Ch_{\left(M\right)}\left[\lambda ,\nu ,t\right]\;=\frac{1}{2}\left(L\left(M,\left(-\lambda +\nu \right),t\right)\;+\;L\left(M,\left(-\lambda -\nu \right),t\right)\right),$$

*and ν is the complex parameter such that*$\nu^{2}=\mu^{2}-\omega^{2}$.

**Proof.**  To get exact expression of the solution for coordinate and momentum operators, we represent the matrix N in the form

(198) $$N=R\;D\;R^{-1},$$

where

(199) $$D\;=\;\left(\begin{array}{ll} -\left(\lambda +\nu \right) & 0\\ 0 & -\left(\lambda -\nu \right) \end{array}\right),$$

(200) $$R\;=\;\left(\begin{array}{ll} -\frac{\mu \;-\;\nu }{c_{-}} & -\frac{\mu \;+\;\nu }{c_{+}}\\ \frac{m\omega^{2}}{c_{-}} & \frac{m\omega^{2}}{c_{+}} \end{array}\right),$$

and

(201) $$c_{\pm }=\sqrt{{\left|\mu \pm \nu \right|}^{2}+{\left(m\omega^{2}\right)}^{2}},\;\nu =\sqrt{\mu^{2}-\omega^{2}}.$$

Using (198), the non-Markovian quantum dynamical map $\Phi_{t}^{\left(M\right)}$ is given as

(202) $$\Phi_{t}^{\left(M\right)}\;=\;L\left(M,N,t\right)\;=\;\sum_{j=1}^{\infty }\left(K\;*\;M^{*,j}\right)\left(t\right)\;{\left(R\;D\;R^{-1}\right)}^{j-1}\;=\;\;\sum_{j=1}^{\infty }\;\left(K*M^{*,j}\right)\left(t\right)\;R\;D^{j-1}\;R^{-1}\;=\;R\;L\left(M,D,t\right)\;R^{-1}.$$

As a result, we have

(203) $$\Phi_{t}^{\left(M\right)}=R\;L\left(M,D,t\right)\;R^{-1}.$$

Substituting expression (199) and (200) into Equation (203), we get the dynamical map

(204) $$\Phi_{t}^{\left(M\right)}\left(\alpha \right)\;=\;\left(\begin{array}{ll} {\mathrm{Ch}}_{\left(M\right)}\left[\lambda ,\nu ,t\right]\;+\;\left(\mu /\nu \right){\;\mathrm{Sh}}_{\left(M\right)}\left[\lambda ,\nu ,t\right] & \left(1/m\nu \right){\;\mathrm{Sh}}_{\left(M\right)}\left[\lambda ,\nu ,t\right]\\ -\left(m\omega^{2}/\nu \right){\;\mathrm{Sh}}_{\left(M\right)}\left[\lambda ,\nu ,t\right] & {\mathrm{Ch}}_{\left(M\right)}\left[\lambda ,\nu ,t\right]\;-\;\left(\mu /\nu \right){\;\mathrm{Sh}}_{\left(M\right)}\left[\lambda ,\nu ,t\right] \end{array}\right),$$

where we use the functions (196) and (197).  □

**Remark** **11.** *For $M\left(t\right)\;=h_{\alpha }\left(t\right)$, we get*

(205) $$\Phi_{t}^{\left(h_{\alpha }\right)}=R\;E_{\alpha }\left[t^{\alpha }D\right]\;R^{-1}.\;$$

*For α=1, map (205) is given by the standard expression*

(206) $$\Phi_{t}^{\left(h_{\alpha }\right)}=\Phi_{t}=R\;e^{tD}\;R^{-1}.\;$$

Theorem 17 describes the non-Markovian dynamics of coordinate and momentum of open quantum system (linear oscillator with friction) with general form on the nonlocality in time.

For λ=0, expressions (194) and (195) describe solutions of non-Markovian generalization of the Heisenberg equation, which is the equation for linear oscillator with Hamiltonian (182) and general form of nonlocality in time.

The solutions of Equations (184) and (185) are described by expressions (194) and (195). Depending on the specific form of the operator kernels $\left(M\left(t\right),K\left(t\right)\right)$, the general trigonometric functions ${\mathrm{Ch}}_{\left(M\right)}$ and ${\mathrm{Sh}}_{\left(M\right)}$, which are given by (196) and (197), will differ. Let us give examples.

**Example** **4.** *For $M\left(t\right)\;=h_{\alpha }\left(t\right)$, the solution of Equations (184) and (185), which describe non-Markovian dynamics of quantum system with power-law memory, were first derived in [37,38,39], where*

(207) $${\mathrm{Sh}}_{\left(M\right)}\left[\lambda ,\nu ,t\right]\;=S_{\alpha }\left[\lambda ,\nu ,t\right]\;=\frac{1}{2}\left(E_{\alpha ,1}\left[\left(-\lambda +\nu \right)t^{\alpha }]\;-\;E_{\alpha ,1}[\left(-\lambda \;-\;\nu \right)t^{\alpha }\right]\right),\;$$

(208) $${\mathrm{Ch}}_{\left(M\right)}\left[\lambda ,\nu ,t\right]\;=C_{\alpha }\left[\lambda ,\nu ,t\right]\;=\frac{1}{2}\left(E_{\alpha ,1}\left(\left(-\lambda +\nu \right)t^{\alpha }\right)\;+\;E_{\alpha ,1}\left(\left(-\lambda -\nu \right)t^{\alpha }\right)\right).$$

*For the case with* α=1 *, equations describe the Markovian dynamics of open quantum systems without nonlocality in time (* α=1 *), since* $E_{1}\left[z\right]\;=exp\left(z\right)$ *, and*

(209) $${\mathrm{Sh}}_{\left(M\right)}\left[\lambda ,\nu ,t\right]\;=e^{-\lambda t}sinh\left(\nu t\right),\;{\mathrm{Ch}}_{\left(M\right)}\left[\lambda ,\nu ,t\right]\;=e^{-\lambda t}cosh\left(\nu t\right),$$

*where* sinh *and* cosh *are hyperbolic sine and cosine.*

**Example** **5.** *For the kernel*

(210) $$M\left(t\right)\;=h_{\alpha }\left(t\right)\;e^{-\beta t}=\frac{t^{\alpha -1}}{\Gamma \left(\alpha \right)}\;e^{-\beta t},\;$$

*where t>0, $\alpha \in \;\left(0,1\right)$, β>0, we have*

(211) $${\mathrm{Sh}}_{\left(M\right)}\left[\lambda ,\nu ,t\right]\;=e^{-\beta t}S_{\alpha }\left[\lambda ,\nu ,t\right]\;+\;\beta \;\int_{0}^{t}d\tau \;e^{-\beta \tau }S_{\alpha }\left[\lambda ,\nu ,\tau \right],$$

(212) $${\mathrm{Ch}}_{\left(M\right)}\left[\lambda ,\nu ,t\right]\;=e^{-\beta t}C_{\alpha }\left[\lambda ,\nu ,t\right]\;+\;\beta \;\int_{0}^{t}d\tau \;e^{-\beta \tau }C_{\alpha }\left[\lambda ,\nu ,\tau \right].$$

**Example** **6.** *For the kernel*

(213) $$M\left(t\right)\;=h_{1-\beta +\alpha }\left(t\right)\;+\;h_{1-\beta }\left(t\right),\;$$

*where 0<α<β<1, we get*

(214) $${\mathrm{Sh}}_{\left(M\right)}\left[\lambda ,\nu ,t\right]\;=\frac{1}{2}(E_{\left(1-\beta ,1-\beta +\alpha \right),1}\left(\left(-\lambda +\nu \right)t^{1-\beta },\;\left(-\lambda +\nu \right)t^{1-\beta +\alpha }\right)\;-\;E_{\left(1-\beta ,1-\beta +\alpha \right),1}\left(\left(-\lambda -\nu \right)t^{1-\beta },\;\left(-\lambda -\nu \right)t^{1-\beta +\alpha }\right)),\;$$

(215) $${\mathrm{Ch}}_{\left(M\right)}\left[\lambda ,\nu ,t\right]\;=\frac{1}{2}(E_{\left(1-\beta ,1-\beta +\alpha \right),1}\left(\left(-\lambda +\nu \right)t^{1-\beta },\left(-\lambda +\nu \right)t^{1-\beta +\alpha }\right)\;+\;E_{\left(1-\beta ,1-\beta +\alpha \right),1}\left(\left(-\lambda -\nu \right)t^{1-\beta },\left(-\lambda -\nu \right)t^{1-\beta +\alpha }\right)),\;$$

*where $E_{\left(1-\beta ,1-\beta +\alpha \right),0}$ is the multinomial Mittag–Leffler function (58).*

## 6. Non-Markovian Quantum Dynamics of Two-Level System

In the case of an N-level open quantum system, the problem was investigated by V. Gorini, A. Kossakowski, and E.C.G. Sudarshan [6]. The general form of the generating superoperator of a completely positive dynamical semi-group of this system has been established [6]. In the case of the N-level quantum system, the Hilbert space H has the dimension dimH=N. Each N-dimensional separable Hilbert space over ℂ is isomorphic to ${\mathbb{C}}^{n}$.

Let us consider a general non-Markovian dynamics of quantum states. The non-Markovian dynamics of the density operator $\rho \left(t\right)$ can be described by the equations with GFD in the form

(216) $$D_{\left(K\right)}^{t,*}\left[\tau \right]\rho \left(\tau \right)\;=\;\frac{1}{i\hbar }\left[H,\rho \left(t\right)\right]\;+\;\frac{1}{\hbar }\sum_{k=1}^{\infty }\left(V_{k}\rho \left(t\right)V_{k}^{†}\;-\;\frac{1}{2}\left\{V_{k}^{†}\;V_{k},\rho \left(t\right)\right\}\right),$$

where $\left\{A,B\right\}\;=A\;B+B\;A$.

Let us consider the non-Markovian two-level quantum systems with general nonlocality in time, which is described by the Sonin kernel $K\left(t\right)$. The Hamiltonian will be considered in the form

(217) $$H=\frac{1}{2}\;\hbar \;\omega_{0}\;\sigma_{3}=\frac{1}{2}\;\hbar \;\omega_{0}\;\left(\left|1\rangle \langle 1\left|\;-\;\right|0\rangle \langle 0\right|\right),$$

where $\omega_{0}>0$ is the transition frequency. Then, the Hamiltonian of the two-level quantum system is diagonal in the basis |0〉, |1〉.

The operators $V_{k}\neq 0$ with k=1,2 will be considered in the form

(218) $$V_{1}=\eta_{1}\;\sigma^{-},\;V_{2}=\eta_{2}\;\sigma^{+},$$

(219) $$V_{1}^{†}=\eta_{1}\;\sigma^{+},\;V_{2}^{†}=\eta_{2}\;\sigma^{-},$$

where $\eta_{1},\eta_{2}\in \mathbb{R}$, and

(220) $$\sigma^{+}\;=\;\left|1\rangle \langle 0\right|\;=\frac{1}{2}\left(\sigma_{1}+i\;\sigma_{2}\right),\;\sigma^{-}\;=\;\left|0\rangle \langle 1\right|\;=\frac{1}{2}\left(\sigma_{1}-i\;\sigma_{2}\right).$$

For operators (217), (218), (219), the general non-Markovian master Equation (216) has the form

(221) $$D_{\left(K\right)}^{t,*}\left[\tau \right]\rho \left(\tau \right)\;=-\frac{i}{\hbar }\left[H,\rho \right]\;+\;\gamma_{1}\left(\sigma^{-}\rho \sigma^{+}-\frac{1}{2}\left\{\sigma^{+}\sigma^{-},\rho \right\}\right)\;+\;\gamma_{2}\left(\sigma^{+}\rho \sigma^{-}-\frac{1}{2}\left\{\sigma^{-}\sigma^{+},\rho \right\}\right),$$

where

(222) $$\gamma_{1}=\frac{1}{\hbar }\eta_{1}^{2},\;\gamma_{2}=\frac{1}{\hbar }\eta_{2}^{2}.$$

We can consider the parameters $\gamma_{1}$ and $\gamma_{2}$ in the form

(223) $$\gamma_{1}=\gamma_{0}\;\left(N_{0}+1\right),\;\gamma_{2}=\gamma_{0}\;N_{0},\;\gamma_{0}=\frac{4\omega_{0}^{3}|d{|}^{2}}{3\hbar c^{3}},$$

where $\gamma_{0}$ is the spontaneous emission rate. Then, Equation (221) with $K\left(t\right)\;=\delta \left(t\right)$ takes the form of Equation (3.219) in [3] (p. 148). In non-Markovian master Equation (221), the terms with $\gamma_{1}$ and $\gamma_{2}$ describe spontaneous emission with rate $\gamma_{0}$, the thermally induced emission and absorption with the rate $\gamma_{0}\;N_{0}$. The total transition rate is equal to

(224) $$\gamma =\gamma_{1}+\gamma_{2}=\gamma_{0}\;\left(2N_{0}+1\right),$$

where $N_{0}=N_{0}\left(\omega_{0}\right)$ is the Planck distribution at the transition frequency.

Using the density operator of two-level system in the form

(225) $$\rho =\sum_{k,l=0}^{1}\rho_{kl}\;\left|k\rangle \langle l\right|,$$

the general non-Markovian equation of this two-level quantum system is represented by the equations with GFD:

(226) $$D_{\left(K\right)}^{t,*}\left[\tau \right]\rho_{00}\left(\tau \right)\;=-\gamma_{2}\rho_{00}+\gamma_{1}\rho_{11}$$

(227) $$D_{\left(K\right)}^{t,*}\left[\tau \right]\rho_{01}\left(\tau \right)\;=i\omega_{0}\rho_{01}-\frac{1}{2}\left(\gamma_{1}+\gamma_{2}\right)\rho_{01}$$

(228) $$D_{\left(K\right)}^{t,*}\left[\tau \right]\rho_{10}\left(\tau \right),=-i\omega_{0}\rho_{10}-\frac{1}{2}\left(\gamma_{1}+\gamma_{2}\right)\rho_{10}$$

(229) $$D_{\left(K\right)}^{t,*}\left[\tau \right]\rho_{11}\left(\tau \right)\;=\gamma_{2}\rho_{00}-\gamma_{1}\rho_{11}$$

The interaction representation of quantum theory cannot be used to solve equations with GFD, which describe non-Markovian dynamics. This fact follows from the violation of the standard Leibniz rule (product rule) for GFD. Note that the time-ordered product (chronological product) for non-Markovian quantum dynamics with nonlocality in type (in the form of memory) was proposed in [45].

To obtain an explicit form of the solution for the components of the density operator of two-level system, Equations (226)–(229) are considered by two pairs.

First pair is the equations

(230) $$D_{\left(K\right)}^{t,*}\left[\tau \right]\rho_{01}\left(\tau \right)\;=\lambda_{1}\;\rho_{01},$$

(231) $$D_{\left(K\right)}^{t,*}\left[\tau \right]\rho_{10}\left(\tau \right)\;=\lambda_{2}\;\rho_{10},$$

where

(232) $$\lambda_{1}=+i\omega_{0}-\frac{1}{2}\left(\gamma_{1}+\gamma_{2}\right),\;\lambda_{2}=-i\omega_{0}-\frac{1}{2}\left(\gamma_{1}+\gamma_{2}\right).$$

For 0<α≤1, the solutions of Equations (230) and (231) have the form

(233) $$\rho_{01}\left(t\right)\;=L\left(M,\lambda_{1},t\right)\;\rho_{01}\left(0\right),$$

(234) $$\rho_{10}\left(t\right)\;=L\left(M,\lambda_{2},t\right)\;\rho_{10}\left(0\right).$$

The second pair is the equations

(235) $$D_{\left(K\right)}^{t,*}\left[\tau \right]\rho_{00}\left(\tau \right)\;=-\gamma_{2}\rho_{00}+\gamma_{1}\rho_{11},$$

(236) $$D_{\left(K\right)}^{t,*}\left[\tau \right]\rho_{11}\left(\tau \right)\;=\gamma_{2}\rho_{00}-\gamma_{1}\rho_{11}.$$

To get solutions of Equations (235) and (236), we considered these equations in the matrix form

(237) $$D_{\left(K\right)}^{t,*}\left[\tau \right]\left(\begin{array}{l} \rho_{00}\left(\tau \right)\\ \rho_{11}\left(\tau \right) \end{array}\right)\;=\;G\;\left(\begin{array}{l} \rho_{00}\\ \rho_{11} \end{array}\right),$$

where

(238) $$G\;=\;\left(\begin{array}{ll} -\gamma_{2} & \gamma_{1}\\ \gamma_{2} & -\gamma_{1} \end{array}\right),$$

and $\gamma_{1}>0$, and $\gamma_{2}>0$.

The solution of Equation (237) has the form

(239) $$\left(\begin{array}{l} \rho_{00}\left(t\right)\\ \rho_{11}\left(t\right) \end{array}\right)\;=S_{t}\left(\alpha \right)\;\left(\begin{array}{l} \rho_{00}\left(0\right)\\ \rho_{11}\left(0\right) \end{array}\right),$$

where

(240) $$S_{t}\left(\alpha \right)\;=L\left(M,G,t\right)\;+\;S\left(\gamma_{1},\gamma_{2}\right),$$

(241) $$S\left(\gamma_{1},\gamma_{2}\right)\;=\;\left(\begin{array}{ll} \gamma_{1}/\gamma & \gamma_{1}/\gamma \\ \gamma_{2}/\gamma & \gamma_{1}/\gamma \end{array}\right)$$

with $\gamma =\gamma_{1}+\gamma_{2}$.

The matrix G can be diagonalized as

(242) $$G\;=\;{\;K}_{1}{\;D}_{1}{\;K}_{1}^{-1},$$

where

(243) $$D_{1}\;=\;\left(\begin{array}{ll} 0 & 0\\ 0 & -\gamma_{1}-\gamma_{2} \end{array}\right),$$

(244) $$K_{1}\;=\;\left(\begin{array}{ll} \frac{\gamma_{1}}{\sqrt{\gamma_{1}^{2}\;+\;\gamma_{2}^{2}}} & -\frac{1}{\sqrt{2}}\\ \frac{\gamma_{2}}{\sqrt{\gamma_{1}^{2}\;+\;\gamma_{2}^{2}}} & \frac{1}{\sqrt{2}} \end{array}\right),$$

(245) $$K_{1}^{-1}\;=\;\left(\begin{array}{ll} \frac{\sqrt{\gamma_{1}^{2}\;+\;\gamma_{2}^{2}}}{\gamma_{1}\;+\;\gamma_{2}} & \frac{\sqrt{\gamma_{1}^{2}\;+\;\gamma_{2}^{2}}}{\gamma_{1}\;+\;\gamma_{2}}\\ -\frac{\sqrt{2}\gamma_{2}}{\gamma_{1}\;+\;\gamma_{2}} & \frac{\sqrt{2}\gamma_{1}}{\gamma_{1}\;+\;\gamma_{2}} \end{array}\right).$$

Exact expression for solution (239) of Equation (237) is derived by the transformations

(246) $$L\left(M,K_{1}{\;D}_{1}{\;K}_{1}^{-1},t\right)\;={\;K}_{1}\;L\left(M,D_{1},t\right){\;K}_{1}^{-1}=$$

(247) $$\left(\begin{array}{ll} \frac{\gamma_{1}}{\sqrt{\gamma_{1}^{2}\;+\;\gamma_{2}^{2}}} & -\frac{1}{\sqrt{2}}\\ \frac{\gamma_{2}}{\sqrt{\gamma_{1}^{2}\;+\;\gamma_{2}^{2}}} & \frac{1}{\sqrt{2}} \end{array}\right)\;\left(\begin{array}{ll} 0 & 0\\ 0 & L\left(M,-\gamma ,t\right) \end{array}\right)\;\left(\begin{array}{ll} \frac{\sqrt{\gamma_{1}^{2}\;+\;\gamma_{2}^{2}}}{\gamma_{1}\;+\;\gamma_{2}} & \frac{\sqrt{\gamma_{1}^{2}\;+\;\gamma_{2}^{2}}}{\gamma_{1}\;+\;\gamma_{2}}\\ -\frac{\sqrt{2}\gamma_{2}}{\gamma_{1}\;+\;\gamma_{2}} & \frac{\sqrt{2}\gamma_{1}}{\gamma_{1}\;+\;\gamma_{2}} \end{array}\right)\;=$$

(248) $$\left(\begin{array}{ll} \frac{\gamma_{2}}{\gamma }\;L\left(M,-\gamma ,t\right) & -\frac{\gamma_{1}}{\gamma }\;L\left(M,-\gamma ,t\right)\\ -\frac{\gamma_{2}}{\gamma }\;L\left(M,-\gamma ,t\right) & \frac{\gamma_{1}}{\gamma }\;L\left(M,-\gamma ,t\right) \end{array}\right).$$

Therefore, we get

(249) $$S_{t}\left(\alpha \right)\;=L\left(M,G,t\right)\;+\;S\left(\gamma_{1},\gamma_{2}\right)\;=\;\left(\begin{array}{ll} \frac{\gamma_{1}}{\gamma }+\frac{\gamma_{2}}{\gamma }\;L\left(M,-\gamma ,t\right) & \frac{\gamma_{1}}{\gamma }-\frac{\gamma_{1}}{\gamma }\;L\left(M,-\gamma ,t\right)\\ \frac{\gamma_{2}}{\gamma }-\frac{\gamma_{2}}{\gamma }\;L\left(M,-\gamma ,t\right) & \frac{\gamma_{2}}{\gamma }+\frac{\gamma_{1}}{\gamma }\;L\left(M,-\gamma ,t\right) \end{array}\right),$$

where $\gamma =\gamma_{1}+\gamma_{2}$, and the solution for $\rho_{00}\left(t\right)$ and $\rho_{11}\left(t\right)$ has in the form

(250) $$\left(\begin{array}{l} \rho_{00}\left(t\right)\\ \rho_{11}\left(t\right) \end{array}\right)\;=\;\left(\begin{array}{ll} \frac{\gamma_{1}}{\gamma }+\frac{\gamma_{2}}{\gamma }\;L\left(M,-\gamma ,t\right) & \frac{\gamma_{1}}{\gamma }-\frac{\gamma_{1}}{\gamma }\;L\left(M,-\gamma ,t\right)\\ \frac{\gamma_{2}}{\gamma }-\frac{\gamma_{2}}{\gamma }\;L\left(M,-\gamma ,t\right) & \frac{\gamma_{2}}{\gamma }+\frac{\gamma_{1}}{\gamma }\;L\left(M,-\gamma ,t\right) \end{array}\right)\cdot \;\left(\begin{array}{l} \rho_{00}\left(0\right)\\ \rho_{11}\left(0\right) \end{array}\right).$$

As a result, we obtain the solution for components of the density matrix $\rho_{kl}\left(t\right)$ in the form

(251) $$\rho_{01}\left(t\right)\;=L\left(M,\lambda_{2},t\right)\;\rho_{01}\left(0\right),$$

(252) $$\rho_{10}\left(t\right)\;=L\left(M,\lambda_{1},t\right)\;\rho_{10}\left(0\right),$$

and

(253) $$\rho_{00}\left(t\right)\;=\;\left(\frac{\gamma_{1}}{\gamma }+\frac{\gamma_{2}}{\gamma }L\left(M,-\gamma ,t\right)\right)\;\rho_{00}\left(0\right)\;+\;\left(\frac{\gamma_{1}}{\gamma }-\frac{\gamma_{1}}{\gamma }L\left(M,-\gamma ,t\right)\right)\;\rho_{11}\left(0\right),$$

(254) $$\rho_{11}\left(t\right)\;=\;\left(\frac{\gamma_{1}}{\gamma }-\frac{\gamma_{2}}{\gamma }L\left(M,-\gamma ,t\right)\right)\;\rho_{00}\left(0\right)\;+\;\left(\frac{\gamma_{1}}{\gamma }+\frac{\gamma_{1}}{\gamma }L\left(M,-\gamma ,t\right)\right)\;\rho_{11}\left(0\right),$$

where $\gamma =\gamma_{1}+\gamma_{2}$.

## 7. Entropy for General Non-Markovian Quantum Dynamics

In quantum mechanics and quantum statistics, the concept of entropy S is defined through the density operator ρ, which is a positive, normalized self-adjoint linear operator. John von Neumann defines [82] the entropy as an extension of the Gibbs entropy concepts from classical mechanics to the quantum mechanics.

For a quantum-mechanical system described by a density operator, the von Neumann entropy is defined (see Sections V.2 and V.3 in [82]) by the equation

(255) $$S\left(t\right)\;=-Tr\left[\rho \left(t\right)\;\ln\rho \left(t\right)\right],$$

where Tr is the trace and ln denotes the (natural) matrix logarithm.

For Markovian dynamics of Hamiltonian quantum systems, the von Neumann entropy does not change

(256) $$S\left(t\right)\;=-Tr\left[\rho \left(t\right)\;\ln\rho \left(t\right)\right]\;=-Tr\left[\rho \left(0\right)\;\ln\rho \left(0\right)\right]\;=S\left(0\right).$$

For non-Markovian and non-Hamiltonian systems, the von Neumann entropy changes in the general case.

Let us consider the von Neumann entropy for general non-Markovian dynamics of two-level quantum systems.

For two-level quantum systems, the density operator is given as

(257) $$\rho \left(t\right)\;=\;\left(\begin{array}{ll} \rho_{00}\left(t\right) & \rho_{01}\left(t\right)\\ \rho_{10}\left(t\right) & \rho_{11}\left(t\right) \end{array}\right).$$

Matrix (257) can be diagonalized

(258) $$\rho \left(t\right)\;=K_{2}{\;D}_{2}{\;K}_{2}^{-1}.$$

The diagonal matrix $D_{2}$ has the form

(259) $$D_{2}\;=\;\left(\begin{array}{ll} D_{-} & 0\\ 0 & D_{+} \end{array}\right),$$

where

(260) $$D_{\pm }=\frac{1}{2}\left(\rho_{00}\left(t\right)\;+\;\rho_{11}\left(t\right)\;\pm \;\sqrt{{\left(\rho_{00}\left(t\right)\;-\;\rho_{11}\left(t\right)\right)}^{2}+4\rho_{01}\left(t\right)\rho_{10}\left(t\right)}\right).$$

Using the normalization condition $Tr\left[\rho \left(t\right)\right]\;=1$ for all $t\in \;\left(0,\infty \right)$, we get

(261) $$\rho_{00}\left(t\right)\;+\;\rho_{11}\left(t\right)\;=\;1.$$

Using representation (258) of density operator (257), we can derive an explicit form of the von Neumann entropy for non-Markovian two-level quantum system.

The von Neumann entropy has the form

(262) $$S\left(t\right)\;=-Tr\left(D_{2}{\;\ln D}_{2}\right),$$

where we use

$$Tr\left[\rho \left(t\right)\ln\left(\rho \left(t\right)\right)\right]\;=Tr\left[K_{2}{\;D}_{2}{\;K}_{2}^{-1}\ln\left(K_{2}{\;D}_{2}{\;K}_{2}^{-1}\right)\right]\;=\;Tr\left[K_{2}{\;D}_{2}{\;K}_{2}^{-1}K_{2}\;\ln\left(D_{2}\right){\;K}_{2}^{-1}\right]\;=\;Tr\left[K_{2}{\;D}_{2}\;\ln\left(D_{2}\right){\;K}_{2}^{-1}\right]\;=Tr\left[K_{2}^{-1}{\;K}_{2}{\;D}_{2}\;\left({\ln D}_{2}\right)\right]\;=Tr\left[D_{2}{\;\ln D}_{2}\right].$$

The von Neumann entropy is given by the equation

(263) $$S\left(t\right)\;=-\frac{1}{2}\left(1-B\left(t\right)\right)\;\ln\left(\frac{1}{2}\left(1-B\left(t\right)\right)\right)\;-\;\frac{1}{2}\left(1+B\left(t\right)\right)\;\ln\left(\frac{1}{2}\left(1+B\left(t\right)\right)\right),$$

where $B\left(t\right)$ is the Bloch vector

(264) $$B\left(t\right)\;=\sqrt{{\left(\rho_{00}\left(t\right)\;-\;\rho_{11}\left(t\right)\right)}^{2}+4\;\rho_{01}\left(t\right)\;\rho_{10}\left(t\right)}.$$

Using solutions (251), (252), and (253), (254), we derive $B\left(t\right)$ in the form

(265) $$B^{2}\left(t\right)\;={\left(\frac{2\gamma_{2}}{\gamma }L\left(M,-\gamma ,t\right)\;\rho_{00}\left(0\right)\;-\;\frac{2\gamma_{1}}{\gamma }L\left(M,-\gamma ,t\right)\;\rho_{11}\left(0\right)\;+\;\frac{\gamma_{1}-\gamma_{2}}{\gamma }\right)}^{2}\;+\;4\;L\left(M,\lambda_{2},t\right)\;L\left(M,\lambda_{1},t\right)\;\rho_{01}\left(0\right)\;\rho_{10}\left(0\right),$$

where $\gamma =\gamma_{1}+\gamma_{2}$ and $\rho_{00}\left(t\right)\;+\;\rho_{11}\left(t\right)\;=1$ for all t≥0 since $Tr\left[\rho \left(t\right)\right]\;=1$.

Let us give some examples of the function $L\left(M,-\gamma ,t\right)$.

(1) For $M\left(t\right)\;=h_{\alpha }\left(t\right)\;=t^{\alpha -1}/\Gamma \left(\alpha \right)$, the second Luchko function has the form
  (266) $$L\left(M,-\gamma ,t\right)\;=E_{\alpha }\left[-\gamma t^{\alpha }\right].$$
(2) For the kernel
  (267) $$M\left(t\right)\;=\frac{t^{\alpha -1}}{\Gamma \left(\alpha \right)}\;e^{-\beta t},$$
  we have
  (268) $$L\left(M,-\gamma ,t\right)\;=\;e^{-\beta t}\;E_{\alpha }\left(-\gamma t^{\alpha }\right)\;+\;\beta \;\int_{0}^{t}d\tau \;e^{-\beta \tau }E_{\alpha }\left(-\gamma \tau^{\alpha }\right),$$
  where $\alpha \in \;\left(0,1\right)$ and β>0.
(3) For the kernel
  (269) $$M\left(t\right)\;=\;\frac{t^{-\beta +\alpha }}{\Gamma \left(1-\beta +\alpha \right)}\;+\;\frac{t^{-\beta }}{\Gamma \left(1-\beta \right)},$$
  the function is
  (270) $$L\left(M,-\gamma ,t\right)\;=\;E_{\left(1-\beta ,1-\beta +\alpha \right),1}\left(-\gamma t^{1-\beta },-\gamma t^{1-\beta +\alpha }\right),$$
  where 0<α<β<1.

## 8. Conclusions

In this paper, we proposed the formulation of non-Markovian quantum theory in the general form. The non-locality in time is represented by kernels of integral and integro-differential operators. These kernels are described by functions that belong to the Sonin set of kernel pairs. The results can be derived in the general form without using special realization of these kernels. Therefore, these results are valid for any operator kernels from the Sonin set. This approach to non-Markovian quantum theory is directly connected with the concept of general fractional dynamics suggested in [72].

Non-Markovian equations for quantum observables and states are suggested by using general fractional calculus. The exact solutions of these equations are derived by using the operational calculus, which is proposed by Luchko in [60] for equations with general fractional derivatives. A wide class of the exactly solvable models of non-Markovian quantum dynamics is suggested. These models describe non-Markovian open quantum systems with the general form of nonlocality in time. The non-Markovian models of quantum oscillator and two-level quantum system with general form of nonlocality in time are described. The exact solutions of equations for these models are proposed.

This paper proposes a general approach to describing non-Markov quantum dynamics. Many important issues are not covered in this article. This work does not offer solutions to all the problems of constructing general non-Markov dynamics of open quantum systems. Let us note some unresolved questions that await their solution in future research.

(1) A quantum system can be embedded in some environments and therefore the system is not isolated. The environment of a quantum system is in principle unobservable or is unknown. This would render the non-Markovian theory of open quantum systems a fundamental generalization of quantum mechanics. However, for practical applications, it is useful to have models of open quantum systems that can be derived from some closed systems including the system under study and some environments. In this regard, the problem arises of constructing models of such closed systems and obtaining general non-Markov dynamics, for example, within the framework of the Caldeira–Leggett approach [83]. At the moment, this problem has not been solved, and the question remains open. We think that the construction of such models is possible. This opinion is based on the following: in the framework of the simplest models, the kernels of fractional derivatives, which describe nonlocality in time, were obtained in [84].
(2) For open quantum systems, its “reduced” dynamics not to violate thermodynamics must not decrease entropy of the evolving state [85]. In this regard, the problem arises of a detailed study of the behavior of entropy for general non-Markov dynamics. At the moment, this problem has not been solved. This question is interesting for further research and computer simulation of the behavior of entropy.
(3) The form of the superoperator L was determined by the Lindblad theorem, which describes the relationship between a completely positive semigroup and a completely dissipative superoperator. The condition for the dissipativity of the superoperator is in fact the standard Leibniz rule, in which equality is replaced by inequality. In non-Markovian dynamics, the semigroup property is violated, and the fractional derivative violates the standard Leibniz rule. In this regard, the question arises about the existence of a generalization of the Lindblad superoperator, in the framework of the proposed general non-Markovian dynamics. In our opinion, such a possibility exists and may be associated with the fractional powers of Lindblad superoperators and the models proposed in the works [5] (pp. 433–444) and [37] (pp. 458–464, 468–477), and [41,42].
